# What are the Differences in Injury Proportions Between Different Populations of Runners? A Systematic Review and Meta-Analysis

**DOI:** 10.1007/s40279-015-0331-x

**Published:** 2015-04-08

**Authors:** Bas Kluitenberg, Marienke van Middelkoop, Ron Diercks, Henk van der Worp

**Affiliations:** Center for Sports Medicine, University Medical Center Groningen, University of Groningen, Hanzeplein 1, 9713 GZ Groningen, The Netherlands; Department of General Practice, Erasmus MC Medical University, Wytemaweg 80, 3015 CN Rotterdam, The Netherlands

## Abstract

**Background:**

Many runners suffer from injuries. No information on high-risk populations is available so far though.

**Objectives:**

The aims of this study were to systematically review injury proportions in different populations of runners and to compare injury locations between these populations.

**Data Sources:**

An electronic search with no date restrictions was conducted up to February 2014 in the PubMed, Embase, SPORTDiscus and Web of Science databases. The search was limited to original articles written in English. The reference lists of the included articles were checked for potentially relevant studies.

**Study Eligibility Criteria:**

Studies were eligible when the proportion of running injuries was reported and the participants belonged to one or more homogeneous populations of runners that were clearly described. Study selection was conducted by two independent reviewers, and disagreements were resolved in a consensus meeting.

**Study Appraisal and Synthesis Methods:**

Details of the study design, population of runners, sample size, injury definition, method of injury assessment, number of injuries and injury locations were extracted from the articles. The risk of bias was assessed with a scale consisting of eight items, which was specifically developed for studies focusing on musculoskeletal complaints.

**Results:**

A total of 86 articles were included in this review. Where possible, injury proportions were pooled for each identified population of runners, using a random-effects model. Injury proportions were affected by injury definitions and durations of follow-up. Large differences between populations existed. The number of medical-attention injuries during an event was small for most populations of runners, except for ultra-marathon runners, in which the pooled estimate was 65.6 %. Time-loss injury proportions between different populations of runners ranged from 3.2 % in cross-country runners to 84.9 % in novice runners. Overall, the proportions were highest among short-distance track runners and ultra-marathon runners.

**Limitations:**

The results were pooled by stratification of studies according to the population, injury definition and follow-up/recall period; however, heterogeneity was high.

**Conclusions:**

Large differences in injury proportions between different populations of runners existed. Injury proportions were affected by the duration of follow-up. A U-shaped pattern between the running distance and the time-loss injury proportion seemed to exist. Future prospective studies of injury surveillance are highly recommended to take running exposure and censoring into account.

**Electronic supplementary material:**

The online version of this article (doi:10.1007/s40279-015-0331-x) contains supplementary material, which is available to authorized users.

## Key Points

**Table:** 

Many studies have examined injury occurrence among runners; however, no information on high-risk populations is available so far.
Large differences in injury proportions existed between different populations of runners.
Injury proportions were affected by the duration of follow-up. Overall, however, time-loss injury proportions were highest among short-distance track runners and ultra-marathon runners.

## Introduction

Injuries are a major problem among runners. Except for previous injuries, no consistent risk factors for running injuries have been found [1]. Running injuries often lead to a reduction in running activity and generally require a long time for recovery [2]. Moreover, injuries are frequently mentioned as a reason for quitting running [3, 4]. Various studies have examined injury proportions (i.e. both incidence proportions and prevalence rates) among runners. The research is, however, characterized by conflicting results, with injury proportions varying between 1.4 [5] and 94.4 % [6]. Several reasons, such as the injury definition, method of injury assessment, study design and follow-up time, form the basis for this lack of consensus. Likewise, the type of runners included in the study sample may play an important role in these conflicting results [7]. It is plausible that injury occurrence and injury type vary between different populations of runners [8]. These differences may explain the large variance in injury proportions observed in running research.

The four-stage injury prevention model developed by Van Mechelen et al. is often used to guide injury prevention research [9]. The first step in this model is to establish the extent of the problem (i.e. the injury incidence). Thereafter, the aetiology of injuries should be studied, and preventive measures can subsequently be introduced. To examine the effectiveness of these interventions, the first stage of the model is repeated [9]. The large variance in injury proportions reported in the literature makes it difficult to determine the extent of the problem. From this perspective, greater insight into the injury susceptibility of different populations of runners may identify specific populations that are at increased risk of sustaining a specific type of injury. This information can be used to assess risk factors for specific high-risk populations, which can be used to develop preventive measures for these populations. A systematic review of the most common injuries in runners demonstrated that injury patterns differed between ultra-marathon runners and runners participating over shorter distances [8]. Until now, however, no systematic review has provided in-depth information on population-specific injury proportions. The primary purpose of this article, therefore, was to systematically review injury proportions in different populations of runners. The secondary objective was to examine differences in injury location between these populations.

## Methods

### Eligibility Criteria and Study Selection

A search with no date restrictions was conducted in the PubMed, Embase, SPORTDiscus and Web of Science databases up to 20 February 2014. The search strategy, as presented in Electronic Supplementary Material Appendix S1, was used to identify articles about injuries in runners. The search was limited to original articles written in English. Studies that met the following eligibility criteria were included in the review:

1. The study design was a prospective cohort study; retrospective cohort study; cross-sectional study; or randomized, controlled trial.
2. The subject of the study was injuries in runners.
3. Injury proportions (incidence proportion or prevalence) were reported.
4. The populations from which the participants were drawn were clearly described.
5. The participants belonged to one or more homogeneous populations of runners, and injury proportions were presented for these different populations separately.
6. Running was the main sport activity of the participants (i.e. not triathlon participants, physical education students or military recruits).

After removal of duplicate studies, all study titles and abstracts were screened by two independent reviewers (BK and HW). All articles of interest were retrieved in full text and evaluated for eligibility by the same independent reviewers. The reference lists of the included articles were checked for other potentially relevant articles that had not been not identified in the electronic search strategy.

Disagreements were resolved in a consensus meeting. On the basis of these articles, nine different populations of interest were defined in which studies were classified (Table 1).

**Table 1:** Definitions of different populations of runners used to classify the articles

Population	Definition
Track: sprinters	Track athletes competing in distances of up to 400 m
Track: middle-distance runners	Track athletes competing in distances of 800–3000 m
Track: long-distance runners	Track athletes competing in 5000 or 10,000 m races
Novice runners	Runners with no regular running experience within the previous year
Recreational runners	Non-competitive runners or runners participating in road races shorter than 10 km
Cross-country runners	Runners competing in cross-country races
Road: long-distance runners	Runners competing in races of between 10 km and less than a marathon
Marathon runners	Runners competing in a marathon
Ultra-marathon runners	Runners competing in races longer than a marathon

### Risk of Bias Assessment and Data Extraction

Risk of bias (ROB) was assessed with a list specifically developed for assessing ROB in studies with different designs focusing on musculoskeletal complaints. The list was developed by van der Windt et al. and was made specific for epidemiology of running injuries by Nielsen et al. [10, 11]. The criteria for the ROB assessment are presented in Electronic Supplementary Material Appendix S2. All items were scored as positive (+) or negative (−) by two independent review authors (BK and HW). When no clear information regarding the item was given or when it was unclear whether the ROB criteria for an item was met, the item was scored as negative. The results of both reviewers’ ROB assessments were compared, and disagreements were resolved in a consensus meeting. The total ROB score for each study was calculated by counting the number of items that were scored positively, expressed as a percentage of all items. Articles with a ROB score ≥50 % were considered as having a low ROB.

From the included articles, descriptive data on the study design, study population, sample size, injury definition, method of injury assessment, number of injuries and injury locations were extracted by one reviewer (BK). When a study reported information for multiple populations of runners, data for each population were extracted separately. Injury definitions were categorized into time-loss injuries, pain-related injuries and medical-attention injuries. When the injury hampered training for at least one day, it was categorized as a time-loss injury. Pain-related injuries were those where running-related pain was assessed regardless of the consequences for running participation (frequency/intensity/duration) and performance. Studies in which runners visited a medical aid station or injuries were reported to a clinician were considered medical-attention injuries.

Details of injury proportions with corresponding follow-up or recall times were acquired from cohort studies. Only injury proportions were extracted from cross-sectional studies. For randomized, controlled trials, the proportion of injuries in the total group of participants was used in the analysis. When information on the anatomical location of the injuries was reported, these data were extracted as well.

### Data Analyses

A meta-analysis was conducted on studies that reported the injury proportion for overall injuries (i.e. studies reporting only a specific injury were excluded from the meta-analysis). First, within each population of runners, studies were categorized into four groups on the basis of the follow-up or recall period during which injuries were monitored. Studies were grouped into studies during an event, studies with a short follow-up/recall period (6–15 weeks), studies with a 1-year follow-up/recall period (11–13 months) and studies with a follow-up/recall period longer than a year. Next, studies with a similar injury definition (i.e. medical-attention, pain-related or time-loss) were grouped. When possible, injury proportions were pooled for each subgroup (same population, same follow-up/recall period and same injury definition) to reduce heterogeneity. R statistics (version 3.1.2; R Core Team 2014) [12] were used to calculate variances around the estimated incidence proportions. The R package meta was used to calculate pooled estimates for all subgroups [13]. Because heterogeneity between studies was expected, random-effect models were used for all analyses. To calculate heterogeneity between studies, *I*^2^ and *τ*^2^ statistics were used. *I*^2^ is an index of heterogeneity and represents the percentage of the total variance that is due to variation between studies, while *τ*^2^ expresses variance between studies in a random-effects meta-analysis [14]. A meta-analysis was first conducted on all studies, followed by a sensitivity analysis on the studies with a low ROB (score ≥50 %).

Site-specific injury proportions were calculated from the extracted data for different anatomical regions. Injuries were categorized into nine different anatomical regions, which were based on another systematic review on injury incidence among long-distance runners (hip/pelvis, upper leg, knee, lower leg, ankle, foot, lower extremity not possible to categorize, not lower extremity and other sites) [15]. For each anatomical location, a weighted average injury proportion was calculated per population of runners.

## Results

### Full-Text Selection

**Fig. 1:**
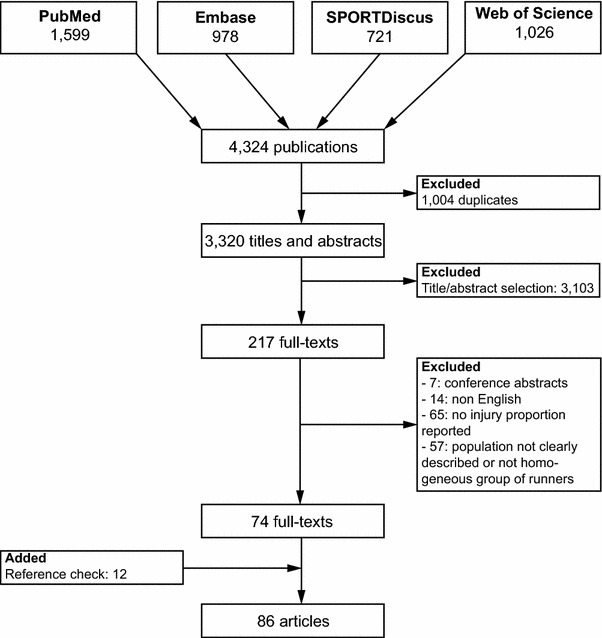
Flow chart of the article selection process

After examination of 3320 titles and abstracts, 217 potentially relevant full-text articles were retrieved. After review of the full texts, 143 articles were excluded. The reference lists of the 74 remaining articles were checked, and 12 articles were added to the review [5, 16–26]. Hence a total of 86 articles were included in the review (Fig. 1).

### Study Characteristics

Fourteen studies presented injury proportions for multiple populations of runners separately [17, 18, 27–38] and were classified into multiple populations of runners (Table 2). For track runners, injury proportions were reported for sprinters in 11 studies [17, 18, 27–32, 39–41], nine studies examined middle-distance runners [17, 18, 27–33] and six studies looked at injury proportions among long-distance track runners [17, 29–33]. Cross-country runners were studied in 21 articles [5, 6, 16, 19–22, 24, 26, 42–53], and long-distance runners were studied in 14 articles [3, 23, 34–38, 54–60]. Most studies were conducted among marathon runners (*N* = 23) [29–31, 34–36, 38, 61–74], while seven studies focused on ultra-marathon runners [37, 75–80]. Thirteen studies monitored injury occurrence among novice runners [25, 81–92]. The smallest number of studies was conducted in recreational non-competitive runners (*N* = 4) [7, 93–95].

**Table 2:** Study characteristics and injury proportions for the different populations of runners

Population and studies	Design	Injury definitions	Injury data	Injury proportions (%)	Time period	ROB (%)
Track: sprinters						
Longo et al. [32]	CS	PRI	AT in 29/41	70.7	–	62.5
Alonso et al. [29]^a^	PC	MA	(1) TLI in 8/327 (2) PRI in 17/327^b^	(1) 2.4 (2) 5.2	9 days (event)	75.0
Alonso et al. [30]	PC	MA	TLI in 16/412	3.9	9 days (event)	62.5
Alonso et al. [31]^a^	PC	MA	(1) TLI in 12/324 (2) PRI in 31/324^b^	(1) 3.7 (2) 9.6	9 days (event)	75.0
Bennell et al. [17]	RC	MA	4 SFs in 2/6	33.3	Lifetime	62.5
Yeung et al. [40]^a^	PC	TLI	(1) Injury in 25/44^b^ (2) 12 hamstring injuries in 8/44	(1) 56.8 (2) 18.2	11 months	87.5
Lysholm and Wiklander [27]^a^	PC	TLI	21 injuries in 13/19^b^; 5.8/1000 h	68.4	1 year	75.0
D’Souza [18]^a^	RC	TLI	Injury in 27/40^b^	67.5	1 year	37.5
Bennell et al. [28]	PC	TLI	4 SFs in 2/16	12.5	1 year	87.5
Sugiura et al. [39]	PC	TLI	Hamstring injury in 6/30	20.0	1 year	75.0
Jacobsson et al. [41]^a^	PC	TLI	122 injuries in 50/77^b^	64.9	1 year	75.0
Track: middle-distance runners						
Longo et al. [32]	CS	PRI	AT in 22/32	68.8	–	62.5
Alonso et al. [29]^a^	PC	MAI	(1) TLI in 5/172 (2) PRI in 16/172^b^	(1) 2.9 (2) 9.3	9 days (event)	75.0
Alonso et al. [30]	PC	MAI–TLI	TLI in 7/202	3.5	9 days (event)	62.5
Alonso et al. [31]^a^	PC	MAI	(1) TLI in 8/154 (2) PRI in 26/154^b^	(1) 5.2 (2) 16.9	9 days (event)	75.0
Bennell at al. [17]	RC	MAI	8 SFs in 5/20	25.0	Lifetime	62.5
Lysholm and Wiklander [27]^a^	PC	TLI	16 injuries in 10/13^b^; 5.6/1000 h	76.9	1 year	75.0
D’Souza [18]^a^	RC	TLI	Injury in 15/27^b^	55.6	1 year	37.5
Bennell et al. [28]	PC	TLI	9 SFs in 8/35	22.9	1 year	87.5
Fredericson et al. [33]	RC	N/A	SF in 20/86	23.3	Lifetime	12.5
Track: long-distance runners						
Longo et al. [32]	CS	PRI	AT in 27/44	61.4	–	62.5
Alonso et al. [29]^a^	PC	MAI	(1) TLI in 6/101 (2) PRI in 17/10^b^	(1) 5.9 (2) 16.8	9 days (event)	75.0
Alonso et al. [30]	PC	MAI–TLI	TLI in 8/130	6.2	9 days (event)	62.5
Alonso et al. [31]^a^	PC	MAI	(1) TLI in 10/105 (2) PRI in 15/105^b^	(1) 9.5 (2) 14.3	9 days (event)	75.0
Bennell et al. [17]	RC	MAI	18 SFs in 6/10	60.0	Lifetime	62.5
Fredericson et al. [33]	RC	N/A	SF in 57/188	30.3	Lifetime	12.5
Novice runners						
Thijs et al. [84]	PC	MAI	PFP in 17/102	16.7	10 weeks	62.5
Ghani Zadeh Hesar et al. [85]	PC	MAI	Injury in 27/131	20.6	10 weeks	62.5
Buist et al. [82, 83]^a^	RCT	TLI	(1) Injury in 48/236; 38/1000 h (2) Injury in 52/250; 30/1000 h (3) Overall: injury in 100/486^b^; 33/1000 h	(1) 20.3 (2) 20.8 (3) 20.6	(1) 8 weeks (2) 13 weeks	62.5 75.0
Bredeweg et al. [88–90]^a^	RCT	TLI	Injury in 58/362^b^; 32/1000 h	16.0	9 weeks	62.5 75.0 50.0
Thijs et al. [87]	PC	MAI	PFP in 1.6/77	20.8	10 weeks	75.0
Van Ginckel et al. [86]^a^	PC	TLI	(1) Injury in 69/129^b^ (2) AT in 10/129	(1) 53.5 (2) 7.8	10 weeks	75.0
Nielsen et al. [92]^a^	PC	TLI	Injury in 13/58^b^	22.4	10 weeks	75.0
Nielsen et al. [25, 91]^a^	PC	TLI	Injury in 254/930^b^	27.3	1 year	75.0
Bovens et al. [81]^a^	PC	TLI	174 injuries in 62/73 runners^b^	84.9	18 months	50.0
Recreational runners						
Lopes et al. [93]	CS	PRI	Injury in 227/1049	21.6	–	62.5
Buist et al. [7]^a^	PC	(1) TLI (2) PRI	(1) TLI in 163/629^b^; 30/1000 h (2) PRI in 217/629^b^	(1) 25.9 (2) 34.5	8 weeks	50.0
Hespanhol Junior et al. [95]^a^	PC	TLI	84 injuries in 60/191^b^; 10/1000 h	31.4	12 weeks	75.0
Hespanhol Junior et al. [94]^a^	RC	TLI	Injury in 110/200^b^	55.0	1 year	62.5
Cross-country runners						
Bennett et al. [53]^a^	(1) RC (2) PC	PRI	(1a) Injury in 26/77 (1b) Injury in 56/77^b^ (2c) Injury in 26/59^b^	(1a) 33.8 (1b) 72.2 (2c) 44.1	(a) 1 month (b) 1 year (c) 1 season	12.5
Beachy et al. [16]^a^	PC	(1) PRI (2) TLI	(1) 843 PRIs in 610/1288^b^ (2) 272 TLIs in 197/1288^b^	(1) 47.4 (2) 15.3	1 season	75.0
Reinking [6]^a^	(1) RC (2) PC	(a) PRI (b) TLI	(1a) PRI in 17/18^b^ (1b) TLI in 12/18^b^ (2a) PRI in 9/18^b^ (2b) TLI in 3/18^b^	(1a) 94.4 (1b) 66.7 (2a) 50.0 (2b) 16.7	(1) Lifetime (2) 1 season	62.5
Plisky et al. [49]	PC	PRI	17 MTSSs in 16/105; 2.8/1000 AEs	15.2	1 season	100
Finnoff et al. [52]	PC	PRI	Knee pain in 3/57	5.3	1 season	62.5
Garrick and Requa [20, 21]^a^	PC	TLI	Injury in 50/167^b^	29.9	1 season	62.5 75.0
Chandy and Grana [5]^a^	PC	TLI	Injury in 31/2278^b^	1.4	1 season	75.0
Rauh et al. [42]^a^	PC	TLI	1622 injuries in 927/3233^b^; 13.1/1000 AEs; 8.7 new injuries/1000 AEs	28.7	1 season	87.5
Rauh et al. [44, 45]^a^	PC	TLI	316 injuries in 162/421^b^; 10.4/1000 AEs	38.5	1 season	87.5 62.5
Reinking and Hayes [46]	(1) RC (2) PC	TLI	(1) Lower leg injury in 33/63 (2) Lower leg injury in 10/32	(1) 52.4 (2) 31.3	(1) HS career (2) 1 season	50.0
Reinking et al. [51]^a^	(1) RC (2) PC	TLI	(1) Injury in 103/125^b^ (2) Injury in 45/93^b^	(1) 82.4 (2) 48.4	(1) Lifetime (2) 1 season	50.0
Grana [22]^a^	PC	TLI	Injury in 9/486^b^	1.9	1 year	25.0
Shively et al. [26]^a^	PC	TLI	Injury in 9/576^b^	1.6	1 year	50.0
McLain and Reynolds [24]^a^	PC	TLI	Injury in 10/94^b^	10.6	1 year	75.0
Laker et al. [48]	CS	N/A	SF in 9/25	36.0	–	25.0
Bennett et al. [43]	PC	N/A	MTSS in 15/125	12.0	1 season	75.0
Reinking et al. [50]	(1) RC (2) PC	N/A	(1) Injury in 60/88 (2) Injury in 26/67	(1) 68.2 (2) 38.8	(1) Running career (2) 1 season	25.0
Kelsey et al. [47]	PC	N/A	SF in 18/127	14.2	2 years	50.0
Eickhoff et al. [19]	RC	N/A	(1) Injury in 101/164 (2) MTSS in 41/164	(1) 61.6 (2) 25.0	Lifetime	37.5
Road: long-distance runners						
Nicholl and Williams [35]^a^	PC	MAI	46 first-aid stops for 44 injuries in 41/1140^b^	3.6	1 day (event)	62.5
Yeung et al. [36]^a^	PC	MAI	Injury in 25/4600^b^	0.5	1 day (event)	62.5
Pasquina et al. [60]^a^	PC	MAI	Injury in 346/91,750^b^	0.4	1 day (event)	62.5
Hughes et al. [54]^a^	RC	TLI	(1) Injury in 725/1266 (2) Injury in 360/1266^b^	(1) 57.3 (2) 28.4	(1) N/A (2) 1 day (event)	62.5
Koplan et al. [23]^a^	RC	TLI	Injury in 498/1423^b^	35.0	1 year	37.5
Marti et al. [57, 58]^a^	RC	TLI	(1) PRI in 2166/4786^b^ (2) TLI in 1372/4786^b^ (3) Severe TLI in 938/4786	(1) 45.3 (2) 28.7 (3) 19.6	1 year	62.5 75.0
Jacobs and Berson [55]^a^	RC	TLI	Injury in 210/451^b^	46.6	2 years	37.5
Koplan et al. [3]^a^	RC	TLI	Injury in 281/535^b^	52.5	10 years	50.0
Lloyd et al. [56]^a^	RC	TLI	Injury in 80/260^b^	30.8	N/A (long)	25.0
Woolf et al. [59]	(1) RC (2) CS	N/A	(1) Low back pain in 327/436 (2) Low back pain in 57/436	(1) 75.0 (2) 13.1	(1) Lifetime (2) –	25.0
Nicholl and Williams [34]	RC	N/A	Injury in 97/242	40.1	1 week	25.0
Micklesfield et al. [37]	RC	N/A	SF in 47/337	13.9	N/A	12.5
Chang et al. [38]	RC	N/A	487 injuries in 334/765	43.7	N/A	12.5
Marathon runners						
Kretsch et al. [63]^a^	(1) CS (2) RC	PRI–MAI	(1) Injury in 151/459 (2a) PRI in 422/459^b^ (2b) MAI in 9/459^b^	(1) 32.9 (2a) 91.9 (2b) 2.0	(1) – (2) 1 day (event)	25.0
Parker et al. [73]^a^	RC	PRI	(1) Injury in 186/374^b^ (2) Injury in 137/374^b^ (3) Injury in 53/374^b^	(1) 49.7 (2) 36.6 (3) 14.2	(1) 1 year (2) N/A (short) (3) 1 day (event)	37.5
Nicholl and Williams [35]^a^	PC	MAI	580 first-aid stops for 534 injuries in 409/2289^b^	17.9	1 day (event)	62.5
Satterthwaite et al. [69, 96]^a^	PC	MAI	(1a) First-aid stops in 75/1219^b^ (1b) 2671 PRIs in 846/916^b^ (2) 1905 PRIs in 723/916	(1a) 6.2 (1b) 92.4 (2) 78.9	(1) 1 day (event) (2) 1 week	75.0 62.5
Yeung et al. [36]^a^	PC	MAI	Injury in 46/900^b^	5.1	1 day (event)	62.5
Roberts et al. [70]^a^	PC	MAI	1534 injuries in 1459/76,714^b^	1.9	1 day (event)	75.0
Ogwumike and Adeniyi [97]^a^	PC	MAI	Injury in 153/920^b^	16.6	1 day (event)	62.5
Alonso et al. [29]^a^	PC	MAI	(1) TLI in 14/151 (2) PRI in 20/151^b^	(1) 9.3 (2) 13.2	9 days (event)	75.0
Alonso et al. [30]	PC	MAI–TLI	TLI in 3/182	1.6	9 days (event)	62.5
Alonso et al. [31]^a^	PC	MAI	(1) TLI in 18/124 (2) PRI in 28/124^b^	(1) 14.5 (2) 22.6	9 days (event)	75.0
Caldwell [61]^a^	RC	TLI	Injury in 68/116^b^	58.3	1 day (event)	25.0
Maughan and Miller [62]^a^	RC	(a) TLI (b) N/A	(1a) TLI in 216/497^b^ (1b) 358 injuries in 287/497 (2b) Injury in 129/449	(1a) 43.5 (1b) 57.7 (2b) 28.7	(1) N/A (short) (2) 1 day (event)	0
Hölmich et al. [65]^a^	RC	TLI	Injury in 26/60^b^ Injury in 7/60^b^	(1) 43.3 (2) 11.7	(1) 1 year (2) 1 day (event)	62.5
Van Middelkoop et al. [71, 72]^a^	(1) RC (2) PC	TLI	(1a) Injury in 397/725^b^ (2b) Injury in 108/725 (2c) Injury in 118/694^b^	(1a) 54.8 (2b) 14.9 (2c) 17.0	1a) 1 year 2b) 1 month 2c) 1 day (event)	75.0 62.5
Rasmussen et al. [74]^a^	RC	TLI	(1) Injury in 273/662^b^ (2) Injury in 68/662^b^	(1) 41.2 (2) 10.3	(1) 1 year (2) 1 day (event)	62.5
Macera et al. [67]^a^	RC	TLI	(1) Injury in 85/162^b^ (2) Injury in 57/162	(1) 52.4 (2) 35.2	(1) 1 year (2) 1 month	50.0
McKelvie et al. [64]^a^	RC	TLI	Injury in 103/126^b^	81.7	12 weeks	25.0
Jakobsen et al. [68]^a^	RCT	TLI	50 injuries in 31/41^b^	75.6	1 year	62.5
Hölmichet al. [66]^a^	RC	TLI	Injury in 410/1310^b^	31.3	N/A (long)	12.5
Nicholl and Williams [34]	RC	N/A	Injury in 203/312	65.1	1 week	25.0
Chang et al. [38]	RC	N/A	117 injuries in 60/125	48.0	N/A (long)	12.5
Ultra-marathon runners						
Hoffman and Krishnan [80]^a^	RC	(1) PRI (2) TLI	(1) 1900 PRIs in 933/1212^b^ (2) TLI in 783/1212^b^	(1) 77.0 (2) 64.6	1 year	25.0
Scheer and Murray [79]^a^	PC	MAI	Clinical encounters in 39/69^b^	56.5	5 days (event)	62.5
Hutson [75]^a^	PC	MAI	31 injuries in 15/25^b^	60.0	6 days (event)	62.5
Bishop and Fallon [77]^a^	PC	MAI	36 injuries in 11/17^b^	64.7	6 days (event)	75.0
Krabak et al. [78]^a^	PC	MAI	1173 injuries in 257/396^b^; 65/1000 h	64.9	7 days (event)	100
Fallon [76]^a^	PC	MAI	64 injuries in 29/32^b^	90.6	8.5 days (event)	50.0
Micklesfield et al. [37]	RC	N/A	SF in 58/276	21.0	N/A	12.5

*AE* athletic exposure, *AT* Achilles tendinopathy, *CS* cross-sectional study, *HS* high school, *MAI* medical-attention injury, *MTSS* medial tibial stress syndrome, *N/A* not available, *PC* prospective cohort study, *PFP* patellofemoral pain, *PRI* pain-related injury, *RC* retrospective cohort study, *RCT* randomized, controlled trial, *ROB* risk of bias, *SF* stress fracture, *TLI* time-loss injury

^a^Study included in the meta-analysis

^b^Data used in the meta-analysis

Of the 86 included articles, 51 were prospective cohort studies [5–7, 16, 20–22, 24–31, 35, 36, 39–47, 49–53, 60, 69–72, 75–79, 81, 84–87, 91, 92, 95–97]. Of these, seven also included a retrospective injury proportion [6, 46, 50, 51, 53, 71, 72]. Twenty-four studies solely used a retrospective cohort design [3, 17–19, 23, 33, 34, 37, 38, 54–58, 61, 62, 64–67, 73, 74, 80, 94]. Five studies reported injury proportions cross-sectionally [32, 48, 59, 63, 93]—two of them retrospectively [59, 63]. From all included studies, nine reported injury incidence densities in addition to injury proportions [7, 27, 42, 44, 49, 78, 83, 88, 95]. Six articles reported the results of a randomized, controlled trial that reported injury occurrence [68, 82, 83, 88–90].

The follow-up periods of the included prospective cohort studies ranged from 1 day to 2 years. For retrospective cohort studies, the recall period varied from 1 day to a lifetime. A time-loss definition was used in 50 studies [3, 5–7, 16, 18, 20–28, 39–42, 44–46, 51, 54–58, 61, 62, 64–68, 71, 72, 74, 80–83, 86, 88–92, 94, 95]. Twenty studies used a medical-attention definition [17, 29–31, 35, 36, 60, 63, 69, 70, 75–79, 84, 85, 87, 96, 97], 11 registered pain-related injuries [6, 7, 16, 32, 49, 52, 53, 63, 73, 80, 93] and in 11 studies, the injury definition was not specified [19, 33, 34, 37, 38, 43, 47, 48, 50, 59, 62]. All study characteristics are presented in Electronic Supplementary Material Appendix S3.

### Risk of Bias

The results of the ROB analysis can be found in Electronic Supplementary Material Appendix S4, and total ROB scores are presented in Table 2. The overall ROB of all included articles was 57.0 %, ranging from 0 to 100 %. Twenty-one articles with an ROB score below 50 were classified as having a high ROB [18, 19, 22, 23, 33, 34, 37, 38, 48, 50, 53, 55, 56, 59, 61–64, 66, 73, 80]. In general, lower ROB scores were the result of low scores on the following items of the ROB checklist: (1) the participation rate was less than 80 %; (2) demographics were missing/incomplete; and (3) the main objective of the study was not to examine injury proportions.

### Meta-analyses of Injury Proportions

**Fig. 2:**
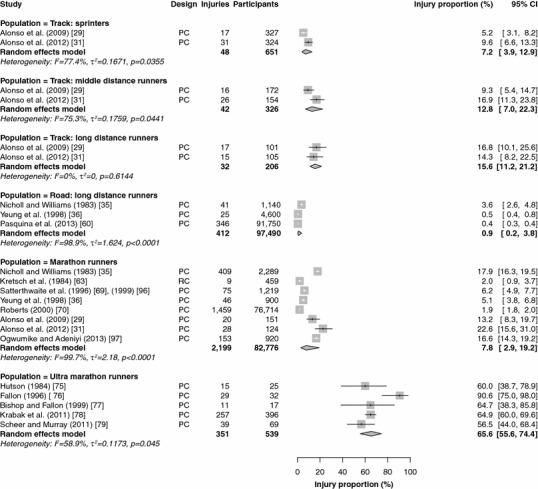
Pooled injury proportions (%) with 95 % confidence intervals (CIs) of participants with injuries requiring medical attention during an event. *PC* prospective cohort study, *RC* retrospective cohort study

**Fig. 3:**
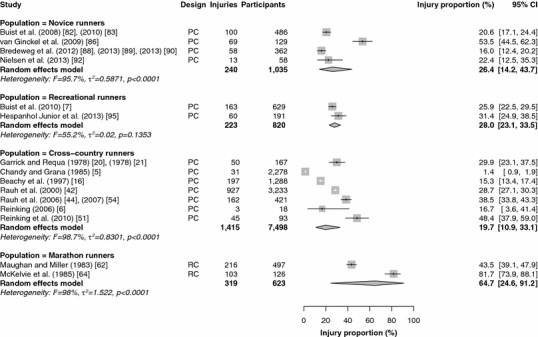
Pooled injury proportions (%) with 95 % confidence intervals (CIs) of participants with time-loss injuries during a short follow-up/recall period. *PC* prospective cohort study, *RC* retrospective cohort study

**Fig. 4:**
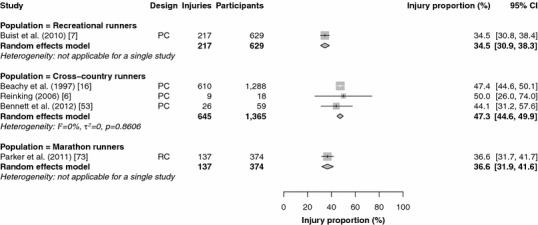
Pooled injury proportions (%) with 95 % confidence intervals (CIs) of participants with pain-related injuries during a short follow-up/recall period. *PC* prospective cohort study, *RC* retrospective cohort study

**Fig. 5:**
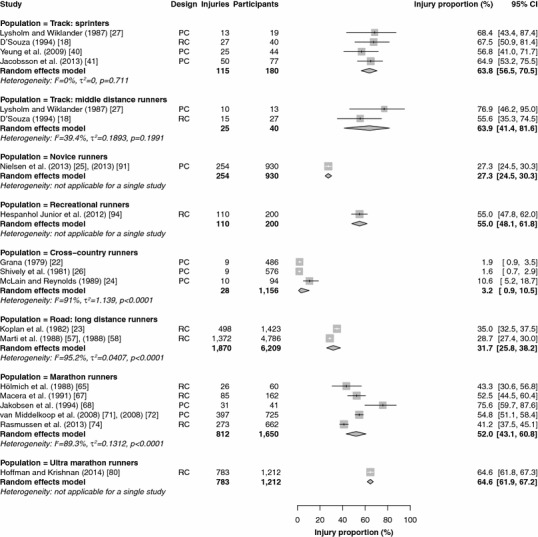
Pooled injury proportions (%) with 95 % confidence intervals (CIs) of participants with time-loss injuries during a 1-year follow-up/recall period. *PC* prospective cohort study, *RC* retrospective cohort study

**Fig. 6:**
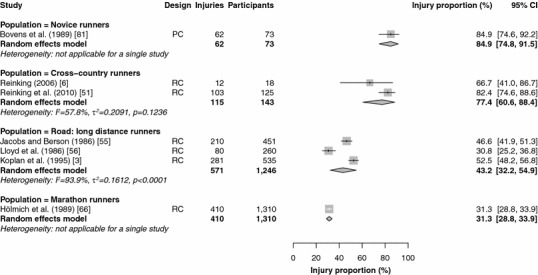
Pooled injury proportions (%) with 95 % confidence intervals (CIs) of participants with time-loss injuries during a follow-up/recall period of >1 year. *PC* prospective cohort study, *RC* retrospective cohort study

Fifteen studies reported injury proportions for specific conditions only [17, 28, 32, 33, 37, 39, 43, 46–49, 52, 59, 84, 87], so the results of those studies could not be pooled (Table 2). The results of the meta-analysis of all studies are shown in Figs. 2, 3, 4, 5 and 6. Heterogeneity was high, as indicated by the *I*^2^ values that exceeded 50 % (Figs. 2, 3, 4, 5 and 6). The results of the sensitivity analysis (ROB ≥50) can be found in Electronic Supplementary Material Appendix S5. The results of the meta-analysis are described below for each follow-up/recall period and injury type.

### Medical-Attention Injuries During an Event

Results were pooled from 22 study populations in which medical encounters during a running event were monitored (Fig. 2) [29, 31, 35, 36, 60, 63, 69, 70, 75–79, 96, 97]. The proportion of medical-attention injuries was highest in ultra-marathon runners [65.6 % (95 % CI 55.6–74.4)] and lowest in road runners [0.9 % (95 % CI 0.2–3.8)]. The injury proportions among elite track runners varied from 7.2 % (95 % CI 3.9–12.9) in sprinters to 12.8 % (95 % CI 7.0–22.3) in middle-distance runners and 15.6 % (95 % CI 11.2–21.2) in long-distance track runners. Medical-attention injuries during an event were not monitored in novice, recreational and cross-country runners. During a marathon race, a medical encounter was registered in 7.8 % (95 % CI 2.9–19.2) of runners. All studies followed runners for a single day, with the exception of most studies in ultra-marathon runners, which followed participants during multi-day competitions. The sensitivity analysis pooled 21 study populations (see Electronic Supplementary Material Appendix S5) [29, 31, 35, 36, 60, 69, 70, 75–79, 96, 97]; this analysis revealed identical results, except for those in marathon runners, who showed an injury proportion that was slightly higher [9.4 % (95 % CI 3.3–23.9)].

#### Time-Loss and Pain-Related Injuries During an Event

Five studies that included only long-distance road runners (*N* = 1) [54] and marathon runners (*N* = 4) [54, 61, 65, 71, 72, 74] assessed the occurrence of time-loss injuries during a race. The data from these studies were pooled. Participants in short road races reported a time-loss injury proportion of 28.4 % (95 % CI 26.0–31.0). Among marathon runners, the pooled time-loss injury proportion was 20.6 % (95 % CI 9.3–39.6). The sensitivity analysis of time-loss injuries during an event consisted of four studies [54, 65, 71, 72, 74]. The injury proportion among long-distance road runners was identical. The pooled injury proportion in marathon runners (*N* = 3) was 13.0 % (95 % CI 8.5–19.3).

Three studies among marathon runners examined the number of pain-related injuries during an event [63, 69, 73, 96]. The pooled estimate was 73.9 % (95 % CI 14.7–97.9). In the sensitivity analysis, one study remained, in which 92.4 % (95 % CI 90.4–94.0) of the participants reported a pain-related injury [69, 96].

#### Time-Loss and Pain-Related Injuries During a Short Follow-Up/Recall Period

Data were pooled from 15 study populations in which time-loss injuries were recorded during a short follow-up/recall period (Fig. 3) [5–7, 16, 20, 21, 42, 44, 45, 51, 62, 64, 82, 83, 86, 88–90, 92, 95]. No studies were conducted in track runners (sprint, middle-distance and long-distance) with a short follow-up/recall period. The pooled injury proportion was highest in marathon runners [64.7 % (95 % CI 25.6–91.2)] and lowest in cross-country runners [19.7 % (95 % CI 10.9–33.1)]. In these studies, cross-country runners were often prospectively followed during a cross-country season (of around 13 weeks).The pooled injury proportions were 26.4 % (95 % CI 14.2–43.7) in novice runners and 28.0 % (95 % CI 23.1–33.5) in recreational runners. Both studies among marathon runners were omitted from the sensitivity analysis [62, 64]; the other results did not change (see Electronic Supplementary Material Appendix S5).

Five studies examined the occurrence of pain-related injuries with a short follow-up/recall period (Fig. 4) [6, 7, 16, 53, 73]. The data from three studies of pain-related injuries among cross-country runners were pooled, resulting in the highest pooled injury proportion [47.3 % (95 % CI 44.6–49.9)] [6, 16, 53]. Pain-related injuries were registered in one study among recreational runners [34.5 % (95 % CI 30.9–38.3)] [7]. In marathon runners, one study monitored pain-related injuries, with an injury proportion of 36.6 % (95 % CI 31.9–41.6) [73]. The study in marathon runners [73] and a study in cross-country runners [53] were excluded from the sensitivity analysis (see Electronic Supplementary Material Appendix S5). The proportions of pain-related injuries among recreational runners were identical in the sensitivity analysis. The estimated injury proportions of pain-related injuries in cross-country runners remained similar [47.4 % (95 % CI 44.7–50.1)].

#### Time-Loss Injuries During a 1-Year Follow-Up/Recall Period

Nineteen studies that monitored time-loss injuries for a 1-year follow-up/recall period were pooled (Fig. 5) [18, 22–27, 40, 41, 57, 58, 65, 67, 68, 71, 72, 74, 80, 91, 94]. The pooled injury proportions were highest in sprinting athletes [63.8 % (95 % CI 56.5–70.5)] and middle-distance track runners [63.9 % (95 % CI 41.4–81.6)]. No data were available for long-distance track runners. The injury proportion was lowest in cross-country runners, at 3.2 % (95 % CI 0.9–10.5). One study that followed novice runners for a year reported a time-loss injury proportion of 27.3 % (95 % CI 24.5–30.3). One study retrospectively assessed 1-year time-loss injury occurrence in recreational runners [55.0 % (95 % CI 48.1–61.8)]. In long-distance road runners and marathon runners, the pooled injury proportions were 31.7 % (95 % CI 25.8–38.2) and 52.0 % (95 % CI 43.1–60.8), respectively. One study reported a 1-year time-loss injury proportion of 64.6 % (95 % CI 61.9–67.2) among ultra-marathon runners. The sensitivity analysis led to small differences in sprinters [62.8 % (95 % CI 54.4–70.4)], middle-distance runners [76.9 % (95 % CI 47.8–92.4)], cross-country runners [4.2 % (95 % CI 0.6–23.9)] and long-distance road runners [28.7 % (95 % CI 27.4–30.0)]. The injury proportions in novice and recreational runners were identical, and there were no studies in ultra-marathon runners (see Electronic Supplementary Material Appendix S5).

#### Time-Loss Injuries During a Long Follow-Up/Recall Period

Results were pooled from seven studies in which time-loss injuries during a long follow-up/recall period were monitored (Fig. 6) [3, 6, 51, 55, 56, 66, 81]. No studies were conducted among sprinting or middle- and long-distance track runners. One study prospectively followed novice runners during an 18-month running programme and reported an injury proportion of 84.9 % (95 % CI 74.8–91.5) [81]. Recreational runners were not studied over periods longer than a year. The pooled injury proportions in cross-country runners and long-distance road runners were 77.4 % (95 % CI 60.6–88.4) and 43.2 % (95 % CI 32.2–54.9), respectively. One study of marathon runners reported an injury proportion of 31.3 % (95 % CI 28.8–33.9) [66]. In the sensitivity analysis, three studies were removed from the analysis (see Electronic Supplementary Material Appendix S5) [55, 56, 66]. This increased the injury proportion among long-distance road runners to 52.5 % (95 % CI 48.3–56.7), and no information on marathon runners was available.

### Anatomical Locations of Injuries

**Fig. 7:**
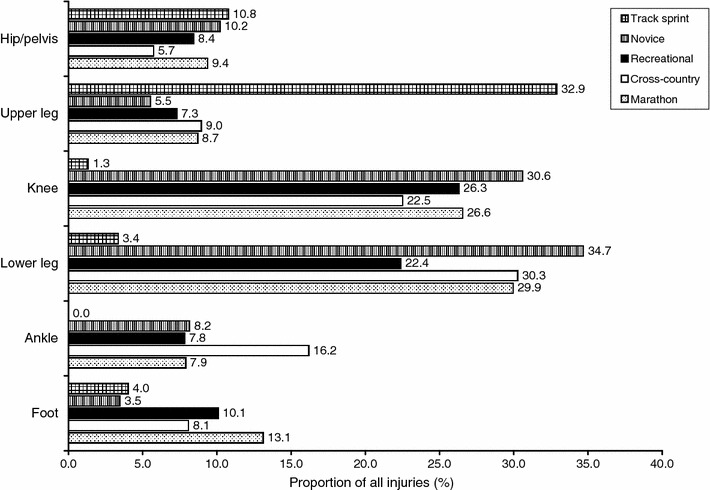
Site-specific time-loss injury proportions (%); injuries during events were excluded. It should be noted that the sum of the injury proportions is not equal to 100 %, because a few injuries could not be classified within these anatomical locations (see Electronic Supplementary Material Appendix S6)

The site-specific injury proportions can be found in Electronic Supplementary Material Appendix S5. An overview of the site-specific time-loss injury proportions can be found in Fig. 7. The numbers of injuries sustained in the hip/pelvis region were similar for all populations of runners, with injury proportions ranging from 5.7 % in cross-country runners to 10.8 % in sprinting track athletes. Injury proportions in the upper leg were small for most populations of runners (5.5–9.0 %). In sprinting athletes, however, most injuries (32.9 %) occurred in the upper leg. The opposite was found for the knee region. Sprinters had the smallest number of injuries in the knee (1.3 %), while the injury proportions in the other populations varied from 22.5 % (in cross-country runners) to 30.6 % (in novice runners). Most injuries in recreational runners were reported around the knee (26.3 %). Novice runners (34.7 %), cross-country runners (30.3 %) and marathon runners (29.9 %) reported the most injuries in the lower leg. Sprinting athletes did not report ankle injuries; this range varied from 7.8 % (in recreational runners) to 16.2 % (in cross-country runners) in the other populations. Novice runners and sprinters reported foot injury proportions of 3.5 and 4.0 %, respectively. For cross-country runners (8.1 %), recreational runners (10.1 %) and marathon runners (13.1 %), the numbers of injuries in the foot were greater.

## Discussion

This is the first systematic review to examine differences in injury proportions between different populations of runners. By pooling injury proportions according to follow-up/recall periods and injury definitions (i.e. time-loss injuries, medical-attention injuries and pain-related injuries) for different populations of runners, we organized the results reported in the literature. Medical-attention injuries were mainly monitored during running events. Except in ultra-marathon runners, the numbers of medical-attention injuries were small in all populations of runners. A few studies monitored pain-related injuries. The highest injury proportion found in this review (94.4 %) was for pain-related injuries among cross-country runners during a lifetime recall period [6]. The lowest injury proportion was found for medical-attention injuries in long-distance runners during an event [60]. Depending on the duration of follow-up/recall, the proportion of time-loss injuries was highest in short-distance track runners (middle-distance runners and sprinters) and ultra-marathon runners.

The numbers of medical-attention injuries during an event varied considerably between different populations of runners. During shorter road races, only 0.9 % of participants requested medical attention at a first-aid station. This percentage was small compared with those in marathon runners (7.8 %) and ultra-marathon runners (65.5 %). This increase in medical attention with increasing running distance could also be seen in track athletes. Only 7.2 % of sprinting athletes required medical attention, while this proportion increased to 15.6 % in long-distance track runners. This discrepancy in injury proportions might have been caused by differences in the accessibility of medical facilities; lack of accessibility is a drawback with regard to registering all problems that require medical attention [98, 99]. This may explain the larger number of medical-attention injuries in marathon runners compared with long-distance road runners, since the number of first-aid stations during a marathon race is relatively large. In a multi-day ultra-race event, runners often had to notify the medical staff about possible problems several times a day. It is likely that this high accessibility of medical services led to the enormous proportion of medical problems reported during ultra-race events. Moreover, accessibility to medical facilities is also dependent on the level of sports participation. For instance, the studies in which medical attention for track runners was registered during an event were all performed in elite athletes during international championships. These athletes have direct access to clinicians, making it impossible to generalize these injury proportions to lower-level track runners. As a result, the reporting of medical-attention injuries does not necessarily reflect the number of injuries sustained in a cohort of runners. Therefore, this method is less suitable for making statements about the number of injuries sustained in a group of runners.

A few studies among marathon runners examined the number of time-loss injuries sustained during an event. The pooled estimate of time-loss injuries during a marathon race was 20.6 %, which is considerably greater than the number of medical-attention injuries reported during a marathon event (7.2 %). Also, 73.9 % of the marathon runners reported pain-related injuries during a race. These differences emphasize that medical-attention injuries do not reflect the number of complaints sustained during an event.

For capturing running-related complaints sustained during a given period of time, time-loss injuries were often registered. When looking at studies with a short follow-up/recall period, no large differences in time-loss injury occurrence existed between novice runners (26.4 %), recreational runners (28.0 %) and cross-country runners (19.7 %). Marathon runners, on the other hand, reported more time-loss injuries during these short periods (64.7 %). These findings suggest that the injury risk is greater for runners training for longer distances. This hypothesis is partly supported by the results from the time-loss injury proportions during a 1-year period. However, middle-distance track runners and sprinters reported high injury proportions too (63.9 and 63.8 %, respectively). Hence, a U-shaped pattern between the running distance and the injury risk may exist. By contrast, the injury proportion reported in cross-country runners was remarkably small (3.2 %). In several studies, injury occurrence was monitored in high school athletes throughout the year [22, 24, 26]. In these studies, injury proportions among cross-country runners varied from 1.6 to 10.6 %, which is relatively small compared with the injury proportion of 19.7 % reported during a single season. One study retrospectively assessed pain-related injuries in cross-country runners over a 1-year period [53], finding an injury proportion of 72.7 %, which indicates that many cross-country athletes suffer from injuries. The exact reason for the small number of time-loss injuries reported among cross-country runners is unknown, but may have to do with the methods of injury surveillance used in the studies among high school athletes. In those studies, injuries were monitored by the coach, who subsequently reported the injuries to the researchers.

In contrast with the studies that had a short or 1-year follow-up/recall period, studies that used a longer period of time displayed an opposite relation between injury proportions and running distance. In these studies, novice runners had the highest injury proportion (84.9 %), followed by cross-country runners (77.4 %). Marathon runners, on the other hand, reported the lowest injury proportions (31.3 %). One notable difference between the study in novice runners and the other studies was its prospective character [81]. More injuries are likely to be registered in a prospective cohort study with a longer follow-up period than in a retrospective study with a similar recall period. It should also be noted that the differences between the populations decreased in the sensitivity analysis, in which only studies with a low ROB were included. This may be an indication that the opposite trend was caused by the small number of high-quality studies that assessed injuries over a longer period of time. After all, only seven studies examined injury occurrence over the long term, and only four of them were suitable for the sensitivity analysis (ROB score ≥50).

In a small number of studies, anatomical regions were registered where the injury occurred. Admittedly, only those studies focusing on all injuries were included in this analysis. The results of the studies showed that in recreational runners, most injuries occurred at the knee, while lower leg injuries were more common in novice, cross-country and marathon runners. This is in line with the review by Lopes et al., which showed that most running injuries were located at the foot, ankle, lower leg and knee [8]. The distribution of injuries across sprinting athletes, however, was notably different: most of their injuries were sustained in the upper leg, followed by the hip/pelvis. During normal running, propulsion is achieved mainly by the structures of the lower leg [100], but during running at high speeds (i.e. sprinting), propulsion is more dependent on power generated at the hip. This is achieved by increasing the demand on the upper leg muscles, resulting in a greater biomechanical load in these structures [100]. This may explain the different injury distribution in sprinting athletes compared with other populations of runners.

It is worth noting that the smallest number of studies involved recreational runners—supposedly the largest group of runners worldwide [95]. This may have to do with the definition of recreational runners that was used in this review (non-competitive runners, or runners participating in road races shorter than 10 km). It is also plausible that this population of runners is ignored for practical reasons. Running events or organized running groups are often used to approach runners for inclusion in a study. The non-organized nature of recreational running makes it difficult to target these runners and include them in a study.

In the literature, it is often assumed that novice runners have a higher injury risk than more experienced runners [7, 82, 101]. The results of this systematic review do not support this assumption, instead giving an indication that the running distance and the injury risk follow a U-shaped pattern, in which short-distance track runners and ultra-marathon runners have the highest injury risk. Unfortunately, most studies included in this review only reported injury proportions over a given period of time. For comparative reasons, it would be better to relate the injury risk to the amount of time spent running [68]—for instance, expressing the number of injuries as a density per 1000 h of running. A number of studies reported injury occurrence in terms of running exposure [7, 27, 42, 44, 49, 78, 83, 88, 95]; their results showed enormous differences between novice runners (33 injuries per 1000 h of running [83]) and middle-distance track runners (5.6 injuries per 1000 h of running [27]). In addition, most studies included in this systematic review did not take censoring into account. Censoring, however, may cause an underestimation of the injury risk in a population of runners. This is particularly true when the cumulative incidence proportion is related to the amount of time spent running. Then, participants who spent less time running would be censored even when they successfully completed the study. In such a case, censoring would result in an increased cumulative injury proportion. In the study by Nielsen et al. among novice runners, an overall injury proportion of 27.3 % was found. However, when censoring was taken into account, the cumulative injury proportion after 500 km of running was almost 50 % [25].

### Limitations

Some limitations of this review should be mentioned. First, only studies written in English were included in the review. Possibly, relevant articles written in other languages were missed. Second, the purpose of this study was to compare injury proportions between different populations of runners. To this end, we identified nine different populations that, in our opinion, discriminated in terms of running experience and running distance. However, the level of running participation was not taken into account. For instance, the studies in track runners included runners participating in world championships, as well as recreational track runners. Third, only studies reporting data in one or more homogeneous populations of runners separately were included in this review. It is therefore possible that some studies were not included, as a result of our defined populations. In addition, the populations might have overlapped; however, by clearly describing our populations, we tried to address this issue. Fourth, injury proportions from studies using a similar injury definition and with a comparable follow-up/recall period were pooled. Definitions were categorized into pain-related injuries, medical-attention injuries and time-loss injuries. There were small differences within injury definitions classified in the same category, which might have influenced the injury proportions reported in these studies. Finally, in this review, both retrospective and prospective cohort studies were pooled. The number of injuries reported in a study is dependent on the study design that is used. It is, however, plausible to assume that this effect becomes more apparent in studies with longer follow-up or recall periods, because of recall bias. In the current review, no distinct differences in injury proportions were observed between prospective and retrospective studies. For this reason, the results of both study designs were pooled. The heterogeneity of the pooled studies was high. This could have been the result of pooling both retrospective and prospective cohort studies. Exclusion of retrospective cohort studies from the meta-analysis did not result in less heterogeneity though. This indicates that the heterogeneity was caused by other differences between studies. Besides differences in populations, study designs and injury definitions, heterogeneity could have been caused by differences in injury assessment. In some studies, injuries were diagnosed by a medical professional, while other injury reports were based on self-reports. Differences in demographics within a population could also lead to heterogeneity. Because injury proportions were the outcome of interest in the current review, a meta-analysis using a random-effects model was used. Consequently, heterogeneity between studies was allowed.

## Conclusions

The numbers of medical-attention injuries during an event were small for most populations of runners, except for ultra-marathon runners, 65.6 % of whom reported medical-attention injuries during a multi-day running event. Large differences in time-loss injury proportions between different populations of runners existed, ranging from a pooled estimate for cross-country runners of 3.2 % to an injury proportion of 84.9 % in novice runners. Injury proportions were affected by the duration of follow-up/recall. Overall, however, a U-shaped pattern between the running distance and the time-loss injury proportion seemed to exist, in which sprinting athletes and ultra-marathon runners had the highest proportions of time-loss injuries. Relatively few studies reported the injury incidence in relation to the amount of time spent running. Future prospective studies of injury surveillance are therefore highly recommended to take running exposure and censoring into account.

## Electronic supplementary material

Supplementary material 1 (PDF 61 kb)

Supplementary material 2 (PDF 63 kb)

Supplementary material 3 (PDF 166 kb)

Supplementary material 4 (PDF 93 kb)

Supplementary material 5 (PDF 618 kb)

Supplementary material 6 (PDF 80 kb)
