# Supplementary material for: What are the Differences in Injury Proportions Between Different Populations of Runners? A Systematic Review and Meta-Analysis
Source: Sports Med. 2015 Apr 8;45(8):1143–61. doi: 10.1007/s40279-015-0331-x (PMC4513221; doi:10.1007/s40279-015-0331-x)
Supplement: Supplementary file 1 — Supplementary material 1 (PDF 61 kb) [file 40279_2015_331_MOESM1_ESM.pdf]

Electronic Supplementary Material Appendix S1. Search strategy that was used for different databases

**Embase**	**Medline/PubMed**	**Web of Science®**	**SPORTDiscus™**
'running'/exp OR (running:ab,ti OR jogg*:ab,ti OR marathon*:ab,ti)	("Running"[Mesh]) OR running[tiab] OR jogg*[tiab] OR marathon*[tiab]	TS=(running OR runner* OR jogg* OR marathon*)	("running"/exp) OR (TI running OR AB running OR TI jogg* OR AB jogg* OR TI marathon* OR AB marathon*)
**AND**			
'injury'/exp OR 'plantar fasciitis'/exp OR (wound*:ab,ti OR injur*:ab,ti OR 'plantar fasciitis':ab,ti)	("Wounds and Injuries"[Mesh]) OR ("Fasciitis, Plantar"[Mesh]) OR "injuries"[sh] OR (wound*[tiab] OR injur*[tiab] OR "plantar fasciitis"[tiab])	TS=(wound* OR injur* OR "plantar fasciitis")	("wounds & injuries"/exp) OR ("plantar fasciitis"/exp) OR (TI wound* OR AB wound* OR TI injur* OR AB injur* OR TI "plantar fasciitis" OR AB "plantar fasciitis")
**AND**			
'epidemiology'/exp OR 'etiology'/exp OR 'risk factor'/exp OR 'cohort analysis'/exp OR (epidemiology:ab,ti OR etiology:ab,ti OR aetiology:ab,ti OR 'risk factor':ab,ti OR 'risk factors':ab,ti OR ‘cohort study’:ab,ti OR 'cohort studies':ab,ti OR incidence:ab,ti OR prevalence:ab,ti)	("Epidemiology"[Mesh]) OR ("Risk Factors"[Mesh]) OR ("Cohort Studies"[Mesh]) OR (epidemiology[sh]) OR (etiology[sh]) OR (epidemiology[tiab] OR etiology[tiab] OR aetiology[tiab] OR "risk factor*"[tiab] OR “cohort study”[tiab] OR “cohort studies”[tiab] OR incidence[tiab] OR prevalence[tiab])	TS=(epidemiology OR etiology OR aetiology OR "risk factor*" OR “cohort study” OR “cohort studies” OR incidence OR prevalence)	("epidemiology"/exp) OR ("diseases -- causes & theories of causation"/exp) OR ("diseases -- risk factors"/exp) OR ("cohort analysis"/exp) OR (TI epidemiology OR AB epidemiology OR TI etiology OR AB etiology OR TI aetiology OR AB aetiology OR TI "risk factor*" OR AB "risk factor*" OR TI "cohort study" OR AB "cohort study" OR TI "cohort studies" OR AB "cohort studies" OR TI incidence OR AB incidence OR TI prevalence OR AB prevalence)
**NOT**		Excluding Document Types=(review)	Lim to (journal article)
[editorial]/lim OR [letter]/lim OR [review]/lim OR 'case report'/de	"case reports"[ptyp] OR "review"[ptyp] OR "editorial"[ptyp] OR "letter"[ptyp]		

Mesh, MH=medical subject heading, sh=subject heading, /exp=explode, *=word truncation, lim=limit, ti=title, ab=abstract, ptyp=publication type
