# Supplementary material for: What are the Differences in Injury Proportions Between Different Populations of Runners? A Systematic Review and Meta-Analysis
Source: Sports Med. 2015 Apr 8;45(8):1143–61. doi: 10.1007/s40279-015-0331-x (PMC4513221; doi:10.1007/s40279-015-0331-x)
Supplement: Supplementary file 2 — Supplementary material 2 (PDF 63 kb) [file 40279_2015_331_MOESM2_ESM.pdf]

**Electronic Supplementary Material Appendix S2.** Checklist for the risk-of-bias assessment

| | |
|---|---|
| *Study objective* | |
| 1 | Positive, if the study had a clearly defined objective and the main purpose was to investigate injury occurrence in runners. |
| *Study population* | |
| 2 | Positive, if the main features of the study population are described (sampling frame and demographic characteristics: gender, age and BMI). |
| 3 | Positive, if the participation rate is at least 80%, or if the participation rate is 60-80% and the non-response is not selective (data shown). |
| 4 | Positive, if the participation rate of the included subjects at main moment of follow-up is at least 80% or if the non-response is not selective (data shown). |
| *Assessment of the outcome* | |
| 5 | Positive, if the time period on which the injury proportion was based is defined or the injury incidence was expressed in terms of exposure. |
| 6 | Method for assessing injury status: physical examination (+); self-reported: specific questions relating to symptoms/disease/use of body chart (+), self-reported: single question (-). |
| 7 | Positive, when the definition of injury was defined. |
| 8 | Positive, if the recall period was less than or equal to a month. |
