# Supplementary material for: What are the Differences in Injury Proportions Between Different Populations of Runners? A Systematic Review and Meta-Analysis
Source: Sports Med. 2015 Apr 8;45(8):1143–61. doi: 10.1007/s40279-015-0331-x (PMC4513221; doi:10.1007/s40279-015-0331-x)
Supplement: Supplementary file 3 — Supplementary material 3 (PDF 166 kb) [file 40279_2015_331_MOESM3_ESM.pdf]

Electronic Supplementary Material Appendix S3. Descriptives of the included studies.

Authors (year)	Population of runners	Study design	Included / analyzed (%)	Description of population	Injury definition	Injury assessment	ROB (%)
Lysholm & Wiklander (1987) [27]	1) Sprint<br>2) Middle	PC	1) 19/19 (100.0)<br>2) 13/13 (100.0)	1) M/F: 12/7<br>Age: 20.6 ± 3.8<br>2) M/F: 9/4<br>Age: 18.6 ± 2.4	All: "Any injuries that markedly hampered training or competition for at least 1 week."	Diagnosis	75.0
D'Souza (1994) [18]	1) Sprint<br>2) Middle	RC	1) 40/40 (100.0)<br>2) 27/27 (100.0)	M/F: 96/51 #<br>Age: 18 ± 2.5 #	All: An injury that lasted more than or is equal to 1 week.	Self-reported	37.5
Bennell et al. (1995) [28]	1) Sprint<br>2) Middle	PC	1) 19/16 (84.2)<br>2) 40/35 (87.5)	M/F: 1) 11/5<br>2) 18/17<br>Age: 20.3 ±2.0 (M); 20.5 ± 2.2 (F) #<br>BMI: 21.8 ± 1.7 (M); 21.1 ± 1.8 (F) #	Stress fractures: "Any musculoskeletal pain or injury that resulted from athletic training and caused alteration of normal training mode, duration, intensity, or frequency for 1 week or more."	Self-reported, Diagnosis	87.5
Bennell et al. (1995) [17]	1) Sprint<br>2) Middle<br>3) Long	RC	1) 6/6 (100.0)<br>2) 20/20 (100.0)<br>3) 10/10 (100.0)	1) M/F: 0/6<br>2) M/F: 0/20<br>3) M/F: 0/10	Stress fractures: "Stress fractures that were diagnosed by a physician based on history and physical examination and confirmed by positive isotope bone scan, radiography, or computerized tomography."	Self-reported, Diagnosis	62.5
Sugiura et al. (2008) [39]	Sprint (elite)	PC	42/30 (71.4)	M/F: 30/0<br>Age: 20.2 ± 1.3<br>BMI: 22.1	Hamstring: An incident of a hamstring injury that caused the sprinter to miss at least 1 week of training or competition.	Diagnosis	75.0
Alonso et al. (2009) [29]	1) Sprint<br>2) Middle<br>3) Long<br>4) Marathon (elite)	PC	1) 327/327 (100.0)<br>2) 172/172 (100.0)<br>3) 101/101 (100.0)<br>4) 151/151 (100.0)	1) M/F: 175/152<br>2) M/F: 88/84<br>3) M/F: 56/45<br>4) M/F: 85/66	All: "All musculoskeletal injuries (traumatic or overuse) newly incurred during competition or training regardless of the consequences with respect to the athlete's absence from competition or training."	Diagnosis	75.0
Longo et al. (2009) [32]	1) Sprint<br>2) Middle<br>3) Long (elite)	CS	178<br>1) 41<br>2) 32<br>3) 44	M/F: 110/68 #<br>Age: 55.4 ± 13.0 (M); 52 ± 9.4 (F) #<br>BMI: 22.9 (M); 21.9 (F) #	Achilles tendinopathy: A score lower than 100 on the Victorian Institute of Sport Assessment-Achilles (VISA-A).	Self-reported	62.5

Yeung et al. (2009) [40]	Sprint	PC	44/44 (100.0)	M/F: 35/9<br>Age: 18.8 (15-24) (M); 19.8 (17-22) (F)<br>BMI: 20.9 (M); 18.6 (F)	1) All: “Missed any practice or competition due to injury of any kind.”<br>2) Hamstring: “Any injury to the hamstring muscle group that occurred as a result of running/sprinting that caused the subject to stop; and the injury prevented the subject from participating in training activities for more than 24 hour.”	1) Self-reported<br>2) Diagnosis	87.5
Alonso et al. (2010) [30]	1) Sprint<br>2) Middle<br>3) Long<br>4) Marathon (elite)	PC	1) 412/412 (100.0)<br>2) 202/202 (100.0)<br>3) 130/130 (100.0)<br>4) 182/182 (100.0)	1) M/F: 239/173<br>2) M/F: 111/91<br>3) M/F: 77/53<br>4) M/F: 102/80	All: “Any musculoskeletal complaint and concussion newly incurred due to competition or training during the study period was regarded as an injury.”	Diagnosis	62.5
Alonso et al. (2012) [31]	1) Sprint<br>2) Middle<br>3) Long<br>4) Marathon (elite)	PC	1) 324/324 (100.0)<br>2) 154/154 (100.0)<br>3) 105/105 (100.0)<br>4) 124/124 (100.0)	1) M/F: 171/153<br>2) M/F: 83/71<br>3) M/F: 62/43<br>4) M/F: 68/56	All: “Any musculoskeletal complaint and concussion newly incurred due to competition or training during the study period was regarded as an injury.”	Diagnosis	75.0
Jacobsson et al. (2013) [41]	Sprint (elite)	PC	108/77 (71.3)	M/F: 59/49<br>Age: 23.4 ± 4.6<br>BMI: 21.5	All: “Any new musculoskeletal pain, soreness or injury that resulted from athletic training or competition and caused changes in normal training/competition to the mode, duration, intensity or frequency from the current or subsequent training and/or competition sessions. Only injuries occurring while participating in athletics, training or competition were included.”	Self-reported	75.0
Fredericson et al. (2005) [33]	1) Middle<br>2) Long	RC	1) 86/86 (100.0)<br>2) 188/188 (100.0)	1) M/F: 32/54<br>Age: 21.6 ± 2.9 (M); 22.2 ± 5.5 (F)<br>2) M/F: 86/102<br>Age: 24.1 (M); 20.9 (F)*	Stress fractures: N/A	Self-reported	12.5
Bovens et al. (1989) [81]	Novice	PC	115/73 (63.5)	M/F: 58/15<br>Age: 35.2 ± 7.9 (M); 33.5 ± 6.4 (F)<br>BMI: 22.7 (M); 21.0 (F)	All: “Any physical complaint developed in relation with running activities and causing restriction in running distance, speed, duration or frequency.”	Self-reported	50.0
Buist et al. (2008, 2010) [82, 83]	Novice	1) RCT<br>2) RCT	603/486 (80.6)	M/F: 226/306 #<br>Age: 39.8 ± 10.1 #<br>BMI: 24.9 ± 2.4 #	Lower extremity: “Running-related musculoskeletal pain of the lower extremity or back causing a restriction of running for at least 1 week.”	Self-reported	1) 62.5<br>2) 75.0

Thijs et al. (2008) [84]	Novice	PC	143/102 (71.3)	M/F: 22/107 #<br>Age: 37 ± 9.5<br>BMI: 25 ± 3	Patellofemoral pain: diagnosis by sports medicine physician.	Diagnosis	62.5
Ghani Zadeh Hesar et al. (2009) [85]	Novice	PC	131/131 (100.0)	M/F: 20/111<br>Age: 39.1 ± 10.3<br>BMI: 24.8	Lower leg overuse: all injuries resulting from participation in the start-to-run program.	Diagnosis	62.5
Van Ginckel et al. (2009) [86]	Novice	PC	129/129 (100.0)	M/F: 19/110<br>Age: 39 ± 10<br>BMI: 24.8 ± 3.5	1) Overuse: "a musculoskeletal ailment that causes a restriction of running speed, distance, duration or frequency for at least 1 week."<br>2) Achilles tendinopathy: N/A	Diagnosis	75.0
Thijs et al. (2011) [87]	Novice	PC	77/77 (100.0)	M/F: 0/77<br>Age: 38 ± 9<br>BMI: 24.6 ± 2.9	1) Lower extremity: "all musculoskeletal injuries of the lower extremities of the study participants who came to seek medical attention."<br>2) Patellofemoral dysfunction syndrome: diagnosis	Diagnosis	75.0
Bredeweg et al. (2012, 2013, 2013) [88-90]	Novice	1) RCT<br>2) RCT<br>3) RCT	500/362 (72.4)	M/F: 149/283 #<br>Age: 38.1 ± 10.8 #<br>BMI: 24.1 ± 3.6 #	Lower extremity: "Any musculoskeletal complaint of the lower extremity or back causing a running restriction for at least 1 week."	Self-reported	1) 62.5<br>2) 75.0<br>3) 50.0
Nielsen et al. (2013, 2013) [25, 91]	Novice	PC	933/930 (99.7)	M/F: 468/461<br>Age: 37.1<br>BMI: 26.3	Lower extremity: "A musculoskeletal complaint of the lower extremity or back caused by running, which restricted the amount of running for at least 1 week. Participant attributed injury (partly) to running."	Diagnosis	1) 75.0<br>2) 75.0
Nielsen et al. (2013) [92]	Novice	PC	118/58 (49.2)	M/F: 32/28<br>Age: 39.8 ± 9.3<br>BMI 25.5 ± 3.9	Lower extremity: "Any musculoskeletal complaint of the lower extremity or back causing a restriction of running for at least 1 week."	Self-reported	75.0
Buist et al. (2010) [7]	Recreational	PC	875/629 (71.9)	M/F: 207/422<br>Age: 43.7 ± 9.5<br>BMI: 24.9 ± 3.3	1) Lower extremity: "Any musculoskeletal pain of the lower limb or back causing a restriction in running (mileage, pace or duration) for at least 1 day."<br>2) Lower extremity: "Running related pain to the lower extremity without restriction of running."	Self-reported	50.0
Lopes et al. (2011) [93]	Recreational	CS	1049	M/F: 796/253<br>Age: 39 ± 11<br>BMI: 24.3 ± 2.8	All: "Current running-related musculoskeletal pain."	Self-reported	62.5
Hespanhol Junior et al. (2012) [94]	Recreational	RC	200/200 (100.0)	M/F: 120/80<br>Age: 43.0 ± 10.5<br>BMI: 24.2 ± 4.3	All: "Any running-related musculoskeletal pain that have being severe enough to prevent the runner to perform at least one training session."	Self-reported	62.5

Hespanhol Junior et al. (2013) [95]	Recreational	PC	200/191 (95.5)	M/F: 141/50<br>Age: 42.8 ± 10.5<br>BMI: 24.4 ± 3.1	All: "Any pain of musculoskeletal origin attributed to running by the runners themselves and severe enough to prevent the runner from performing at least one training session."	Self-reported	75.0
Garrick & Requa (1978, 1978) [20, 21]	Cross	PC	167/167 (100.0)	M/F: 141/26	All: "Medical problems arising as a direct result of interscholastic sport participation that required a participant to be withdrawn from a practice or competitive event or miss participation in a subsequent practice or competitive event."	Coach reported	1) 62.5<br>2) 75.0
Grana (1979) [22]	Cross	PC	486/486 (100.0)	N/A	All: "Any event that altered the participant's ability to participate in a game or practice in the usual manner of other participants."	Coach reported	25.0
Shively et al. (1981) [26]	Cross	PC	576/576 (100.0)	M/F: 389/187	All: "Any event that altered the ability of a participant to compete or practice in the usual manner."	Coach reported	50.0
Chandy & Grana (1985) [5]	Cross	PC	2,278/2,278 (100.0)	M/F: 1,567/711	All: "Any problem that resulted in an altered or lost practice session or game."	Coach reported	75.0
McLain & Reynolds (1989) [24]	Cross	PC	94/94 (100.0)	M/F: 54/40	All: "Any incident resulting from athletic participation that keeps an athlete from completing a practice or game, or causes the athlete to miss a subsequent practice or game."	Coach reported	75.0
Beachy et al. (1997) [16]	Cross	PC	1,288/1,288 (100.0)	M/F: 501/787	1) All: "Any athlete complaint that required the attention of the athletic trainer, regardless of the time lost from activity."<br>2) All: "All injuries reported to the athletic training staff that resulted in at least 1 day of lost activity."	Coach reported	75.0
Eickhoff et al. (2000) [19]	Cross	RC	164/164 (100.0)	M/F: 91/73	Lower leg: "A lower leg injury as a result of running."<br><br>Medial tibial stress syndrome: "Pain is most commonly found on the medial border of the tibia or the inside of the lower leg bone closest to the middle of the body. The pain during activity will increase but may decrease when not weight-bearing. There may be some swelling associated with the lower leg pain in the medial muscle area next to the middle leg bone of the lower leg."	Self-reported	37.5
Rauh et al. (2000) [42]	Cross	PC	3,233/3,233 (100.0)	M/F: 2,031/1,202	All: "A medical problem resulting from athletic participation that required an athlete to be removed from a practice or competitive event or to miss a subsequent practice or competitive event."	Coach reported	87.5

Bennett et al. (2001) [43]	Cross	PC	125/125 (100.0)	M/F: 57/68<br>Age: 16.0 ± 1.1	Medial tibial stress syndrome: “Athletes had symptoms of medial tibial stress syndrome and had pain in the symptomatic limb with palpation along the medial border.”	Coach reported, Diagnosis	75.0
Rauh et al. (2006, 2007) [44, 45]	Cross	PC	1) 421/421 (100.0)<br>2) 421/393 (93.3)	M/F: 235/186<br>BMI: 20.4 ± 2.0	Lower extremity: “Any reported muscle, joint, or bone problem/injury of the back or lower extremity resulting from running in a practice or meet and requiring the runner to be removed from a practice or meet or to miss a subsequent one.”	Coach reported	1) 87.5<br>2) 62.5
Reinking (2006) [6]	Cross	1) PC<br>2) RC	18/18 (100.0)	M/F: 0/18<br>Age: 19.5 ± 1.2<br>BMI: 21.1 ± 1.8	Lower extremity: “Athletes who rated their Exercise Related Leg Pain as Nirschl 2 or greater.”<br>1) Without interference in sports participation<br>2) Interfering with sports participation	Self-reported, Diagnosis	62.5
Reinking & Hayes (2006) [46]	Cross	1) RC<br>2) PC	1) 63/63 (100.0)<br>2) 32/32 (100.0)	M/F: 30/33<br>Age: 19.4 ± 1.2	Lower leg: “Pain between their knee and ankle associated with exercise that had interfered with their cross country training or competition.”	1) Self-reported<br>2) Coach reported	50.0
Kelsey et al. (2007) [47]	Cross	PC	127/127 (100.0)	M/F: 0/127<br>Age: 22.0 ± 2.6<br>BMI: 21.2 ± 1.9	Stress fractures: N/A	Self-reported, Diagnosis	50.0
Laker et al. (2007) [48]	Cross	CS	25	M/F: 15/10	Stress fractures: N/A	Diagnosis	25.0
Plisky et al. (2007) [49]	Cross	PC	130/130 (100.0)	M/F: 59/46<br>Age: 15.0 ± 1.0<br>BMI: 20.5	Medial Tibial Stress Syndrome: “Continuous or intermittent pain in the tibial region, exacerbated with repetitive weight-bearing activity, and localized pain with palpation along the distal two thirds of the posterior-medial tibia.”	Coach reported	100.0
Reinking et al. (2007) [50]	Cross	1) RC<br>2) PC	1) 88/88 (100.0)<br>2) 88/67 (76.1)	M/F: 44/44<br>Age: 19.5 (18-24)	Lower extremity: N/A	Self-reported	25.0
Reinking et al. (2010) [51]	Cross	1) RC<br>2) PC	125/93 (74.4)	M/F: 63/62<br>Age: 15.4 (13-18)	Lower extremity: Whether excersise related lower leg pain had caused the athlete to miss at least 1 day of training, or whether excersise related lower leg pain had negatively affected the athlete’s race time.”	Self-reported	50.0
Finnoff et al. (2011) [52]	Cross	PC	57/57 (100.0)	M/F: 53/44 #<br>Age: 16.0 (14-18)*	Knee pain: Noble compression test, patellar grind test	Diagnosis	62.5
Bennett et al. (2012) [53]	Cross	1) RC<br>2) PC	77/59 (76.6)	M/F: 44/33	Lower extremity: “Pain located in the anterior, medial, posterior, or lateral leg not associated with a traumatic injury.”	Self-reported	12.5
Koplan et al. (1982) [23]	Road	RC	2,500/1,423	M/F: 693/730<br>Age: 33.4 (M); 29.9 (F)	All: “A musculoskeletal ailment attributed to running that caused the runner to reduce the weekly mileage, take medicine, or visit a health professional.”	Self-reported	37.5

Nicholl & Williams (1982) [34]	1) Road<br>2) Marathon	RC	614/554 (90.2)<br>1) 242<br>2) 312	1) M/F: 178/64<br>Age: 180 < 40 years<br>62 ≥ 40 years<br>2) M/F: 283/29<br>Age: 239 < 40 years<br>73 ≥ 40 years	All: N/A	Self-reported	25.0
Nicholl & Williams (1983) [35]	1) Road<br>2) Marathon	PC	1) 1,140/1,140 (100.0)<br>2) 2,289/2,289 (100.0)	M/F: 3,264/198 #<br>Age:<br>304 between 18-29<br>150 between 30-39<br>63 ≥ 40	All: visit to the first-aid post during the competition.	Diagnosis	62.5
Hughes et al. (1985) [54]	Road	RC	1,266/1,266 (100.0)	M/F: 1,078/188<br>Age 32.3 (9-75)	All: "Orthopedic problems or any specific running injuries severe enough to interfere with their training schedules."	Self-reported	62.5
Jacobs & Berson (1986) [55]	Road	RC	487/451 (92.6)	M/F: 355/96<br>Age: 33.9 (14-64) (M); 32.4 (8-57)	Lower extremity: "Any lower extremity or lower back pain that caused a restriction in running, distance or speed, or prevented any running at all."	Self-reported	37.5
Lloyd et al. (1986) [56]	Road	RC	267/260 (97.4)	M/F: 0/260<br>Age: 32.2 ± 0.7<br>BMI: 20.9	All: "Any musculoskeletal ailment attributed to running that caused the runner to interrupt her running program and/or to seek medical help."	Self-reported	25.0
Marti et al. (1988, 1988) [57, 58]	Road	RC	5,534/4,786 (86.5)	M/F: 4,358/428<br>Age: 34.9 (M), 32.6 (F)<br>BMI: 22.4 ± 1.9 (M); 20.3 ± 1.6 (F)	1) All: injuries involved maintenance of full training activity in spite of symptoms (grade 1)<br>2) All: injuries involved a reduction of training activity (grade 2)<br>3) All: injuries involved full training interruption of at least 2 weeks (grade 3)	Self-reported	1) 62.5<br>2) 75.0
Koplan et al. (1995) [3]	Road	RC	742/535 (72.1)	M/F: 326/209<br>Age:<br>59 ≤ 29 years<br>113 between 30-39<br>227 between 40-49<br>103 between 50-59<br>32 ≥ 60 years<br>BMI: 24.6 (M); 21.5 (F)	All: "A musculoskeletal ailment that caused respondents to reduce or cease exercise or interfered with their work or school activities."	Self-reported	50.0
Yeung et al. (1998) [36]	1) Road<br>2) Marathon	PC	1) 4,600/4,600 (100.0)<br>2) 900/900 (100.0)	N/A	All: "All runners who required physiotherapy services"	Diagnosis	62.5
Woolf et al. (2002) [59]	Road	1) RC<br>2) CS	436/436 (100.0)	M/F: 52% / 48%<br>Age: 39.4 (12-73) (M); 33.5 (11-65) (F) #	Low back pain: N/A	Self-reported	25.0

Micklesfield et al. (2007) [37]	1) Road<br>2) Ultra	RC	1) 337/337 (100.0)<br>2) 276/276 (100.0)	1) M/F: 0/337<br>Age: 34 ± 10.5<br>BMI: 21.9 ± 2.9<br>2) M/F: 0/276<br>Age: 39 ± 8.2<br>BMI: 21.6 ± 2.4	Stress fractures: N/A	Self-reported	12.5
Chang et al. (2012) [38]	1) Road<br>2) Marathon	RC	1,004/893 (88.9)<br>1) 765<br>2) 128	1) M/F: 668/97<br>Age: 33.6 ± 9.8 #<br>BMI: 22.9 #<br>2) M/F: 122/6<br>Age: 38.8 ± 11.6<br>BMI: 22.4	Lower extremity: N/A	Self-reported	12.5
Pasquina et al. (2013) [60]	Road	PC	91,750/91,750 (100.0)	M/F: 48% / 52%<br>Age: 64% within 25-44	All: participants treated by medical personnel	Diagnosis	62.5
Caldwell (1981) [61]	Marathon	RC	302/84 (27.8)	M/F: 269/42<br>Age: 12-62	All: "Any physical problem that the runner felt negatively affected his/her performance."	Self-reported	25.0
Maughan & Miller (1983) [62]	Marathon	RC	497/449 (90.3)	M/F: 472/25<br>Age: 32 ± 8	1) All: injuries occurred during preparation for the race.<br>2) All: injuries occurred during the race.	Self-reported	0.0
Kretsch et al. (1984) [63]	Marathon	1) RC<br>2) CS	1,098/459	M/F: 348/111	All: "Injuries occurring immediately before the race; medical problems experienced during the race; pains or 'unusual' symptoms developing after the race."	Self-reported, Diagnosis	25.0
McKelvie et al. (1985) [64]	Marathon	RC	127/126 (99.2)	M/F: 105/21<br>Age: 34.9 ± 9.1 (M)<br>BMI: 22.4 (M)	All: interruptions of training due to injury.	Self-reported	25.0
Hölmich et al. (1988) [65]	Marathon	RC	60/60 (100.0)	M/F: 60/0<br>Age: 28 (18-51)	All: Injury preventing them from training.	Self-reported	62.5
Hölmich et al. (1989) [66]	Marathon	RC	1,426/1,310 (91.9)	M/F: 1,310/0<br>Age: 34 (11-77)<br>BMI: 22.9	All: Injury preventing them from training or work.	Self-reported	12.5
Macera et al. (1991) [67]	1) Marathon	RC	964/509 (52.8) #<br>1) 162	M/F: 146/16<br>Age: 36 (M); 33 (F)	Lower extremity: "Problems in any of the following sites (foot, ankle, Achilles tendon, calf or shin, knee, thigh, and hip), regardless of cause, that required a consultation with a physician or reduction in usual running mileage."	Self-reported	50.0
Jakobsen et al. (1994) [68]	Marathon	RCT	41/41 (100.0)	M/F: 37/4<br>Age: 41.8 *<br>BMI: 22.7 *	All: "Any injury of the musculoskeletal system that was sustained during running and prevented training or competition."	Self-reported	62.5

Satterthwaite et al. (1996, 1999) [69, 96]	Marathon	PC	1,054/916 (86.9)	M/F: 80.3% / 19.7%<br>Age: 38.6 ± 9.8	1) All: Specific health problems, using a matrix of 13 body sites by 11 problem types.<br>2) All: Injuries and other health problems sustained by runners attending a medical aid post.	1) Self-reported<br>2) Diagnosis	1) 75.0<br>2) 62.5
Roberts (2000) [70]	Marathon	PC	76,714/76,714 (100.0)	M/F: 59,751/16,963 #	All: "Any disability that interferes with function where medical assistance is either requested or rendered at the finish line medical tent."	Diagnosis	75.0
Van Middelkoop et al. (2008, 2008) [71, 72]	Marathon	1) RC<br>2) PC	1) 726/725 (99.9)<br>2) 724/694 (95.9)	M/F: 725/0<br>Age: 43.7 ± 9.6<br>BMI: 23.6 ± 2.1	Lower extremity: "An injury to muscles, joints, tendons, and/or bones of the lower extremities that the participant attributed to running. The problem had to be severe enough to cause a reduction in distance, speed, duration, or frequency of running."	Self-reported	1) 75.0<br>2) 62.5
Parker et al. (2011) [73]	Marathon	RC	545/374 (68.6)	M/F: 0/378	All: Injuries leading to: No change in running pace or routine or slowed pace while running, same weekly routine or slowed pace while running and decreased weekly mileage or stopped running for less than 1 week or stopped running for 1 to 4 weeks or stopped running for more than 1 month.	Self-reported	37.5
Rasmussen et al. (2013) [74]	Marathon	RC	680/662 (97.4)	M/F: 535/127<br>Age: 41.4 ± 10.4<br>BMI: 23.0 ± 2.3	All: "An injury to muscles, tendons, joints and/or bones caused by running. The injury had to be severe enough to cause or be expected to cause a reduction in distance, speed, duration, or frequency of running for at least 14 days."	Self-reported	62.5
Ogwumike & Adeniyi (2013) [97]	Marathon	PC	920/920 (100.0)	M/F: 856/64<br>Age: 24.8 *	All: runners who contacted the paramedical services.	Diagnosis	62.5
Hutson (1984) [75]	Ultra	PC	25/25 (100.0)	M/F: 23/2<br>Age: 41 (23-57)	All: An injury sufficiently severe to impair their performance.	Diagnosis	62.5
Fallon (1996) [76]	Ultra	PC	32/32 (100.0)	Age: 38 (23-53) (M);<br>43 (40-46) (F)	All: "An injury was recorded only following examination of the runner after a request from the runner or crew, or following a decision by the medical staff member."	Diagnosis	50.0
Bishop & Fallon (1999) [77]	Ultra	PC	17/17 (100.0)	M/F: 16/1<br>Age: 47 ±f 11	All: "A specific musculoskeletal abnormality that the runner perceived to be affecting his or her performance. Conditions ubiquitous in ultra-marathon, such as muscle soreness, cramps, and blisters were not included."	Diagnosis	75.0
Krabak et al. (2011) [78]	Ultra	PC	396/396 (100.0)	M/F: 79.2% / 20.8%<br>BMI: 23.9 ± 3.5	All: "A disability sustained by a study participant during the race, resulting in a medical encounter by the medical staff."	Diagnosis	100.0

Scheer & Murray (2011) [79]	Ultra	PC	69/69 (100.0)	M/F: 48/21<br>Age: 46 (27-63) (M); 40 (26-50)	All: "All clinical encounters from the start of the first stage until the clinic immediately after the last stage."	Diagnosis	62.5
Hoffman & Krishnan (2014) [80]	Ultra	RC	1,212/1,212 (100.0)	M/F: 68% / 32%<br>Age: 42.3 (18-81)	1) All: exercise-related injuries without training loss.<br>2) All: exercise-related injuries with at least one day training loss.	Self-reported	25.0

PC: Prospective Cohort, RC: Retrospective Cohort, RCT: Randomized Controlled Trial, CS: Cross-Sectional, ROB: Risk of Bias, M: Male, F: Female, N/A: Not Available
#: Descriptives for total population or subgroup, instead of study sample.
*: Mean is calculated based on different groups.
