# Supplementary material for: What are the Differences in Injury Proportions Between Different Populations of Runners? A Systematic Review and Meta-Analysis
Source: Sports Med. 2015 Apr 8;45(8):1143–61. doi: 10.1007/s40279-015-0331-x (PMC4513221; doi:10.1007/s40279-015-0331-x)
Supplement: Supplementary file 4 — Supplementary material 4 (PDF 93 kb) [file 40279_2015_331_MOESM4_ESM.pdf]

Electronic Supplementary Material Appendix S4. Risk of Bias scores of the included articles.

Study	Criteria for Risk of Bias assessment (Electronic Supplementary Material Appendix S2)								
	1	2	3	4	5	6	7	8	Score
**Track: sprinters**									
Lysholm and Wiklander [27]	+	-	-	+	+	+	+	+	75.0%
D'Souza [18]	+	-	+	-	+	-	-	-	37.5%
Bennell et al. [28]	+	+	-	+	+	+	+	+	87.5%
Bennell et al. [17]	+	+	-	+	-	+	+	-	62.5%
Sugiura et al. [39]	-	+	-	+	+	+	+	+	75.0%
Alonso et al. [29]	+	-	+	+	+	+	-	+	75.0%
Longo et al. [32]	-	+	-	+	-	+	+	+	62.5%
Yeung et al. [40]	+	+	-	+	+	+	+	+	87.5%
Alonso et al. [30]	+	-	-	+	+	+	-	+	62.5%
Alonso et al. [31]	+	-	+	+	+	+	-	+	75.0%
Jacobsson et al. [41]	+	+	-	-	+	+	+	+	75.0%
**Track: middle-distance runners**									
Fredericson et al. [33]	-	-	-	+	-	-	-	-	12.5%
**Novice runners**									
Bovens et al. [81]	-	+	-	-	+	-	+	+	50.0%
Buist et al. [82]	+	+	-	+	+	-	+	-	62.5%
Thijs et al. [84]	-	+	-	-	+	+	+	+	62.5%
Ghani Zadeh Hesar et al. [85]	-	+	-	+	+	+	-	+	62.5%
van Ginckel et al. [86]	-	+	-	+	+	+	+	+	75.0%
Buist et al. [83]	-	+	-	+	+	+	+	+	75.0%
Thijs et al. [87]	-	+	-	+	+	+	+	+	75.0%
Bredeweg et al. [88]	+	+	-	+	+	-	+	-	62.5%
Bredeweg et al. [89]	-	+	-	+	+	+	+	+	75.0%
Bredeweg et al. [90]	-	+	-	+	+	-	+	-	50.0%
Nielsen et al. [25]	-	+	-	+	+	+	+	+	75.0%
Nielsen et al. [91]	-	+	-	+	+	+	+	+	75.0%
Nielsen et al. [92]	-	+	-	+	+	+	+	+	75.0%
**Recreational runners**									
Buist et al. [7]	+	+	-	-	+	-	+	-	50.0%
Lopes et al. [93]	+	+	-	-	-	+	+	+	62.5%
Hespanhol Junior et al. [94]	+	+	-	-	+	+	+	-	62.5%
Hespanhol Junior et al. [95]	+	+	-	-	+	+	+	+	75.0%
**Cross-country runners**									
Garrick and Requa [21]	-	-	-	+	+	+	+	+	62.5%
Garrick and Requa [20]	+	-	-	+	+	+	+	+	75.0%
Grana [22]	-	-	-	-	+	-	+	-	25.0%
Shiveley et al. [26]	-	-	-	+	+	+	+	-	50.0%
Chandy and Grana [5]	+	-	+	+	+	-	+	+	75.0%
McLain and Reynolds [24]	-	-	+	+	+	+	+	+	75.0%
Beachy et al. [16]	+	-	-	+	+	+	+	+	75.0%
Eickhoff et al. [19]	+	-	-	-	-	+	+	-	37.5%
Rauh et al. [42]	+	-	+	+	+	+	+	+	87.5%
Bennett et al. [43]	+	-	+	+	-	+	+	+	75.0%
Rauh et al. [44]	+	-	+	+	+	+	+	+	87.5%
Reinking [6]	-	+	+	+	-	+	+	-	62.5%
Reinking and Hayes [46]	+	-	-	+	-	+	+	-	50.0%
Kelsey et al. [47]	-	+	-	+	-	+	-	+	50.0%
Laker et al. [48]	-	-	-	-	-	+	-	+	25.0%
Plisky et al. [49]	+	+	+	+	+	+	+	+	100.0%
Rauh et al. [45]	-	-	+	+	-	+	+	+	62.5%
Reinking et al. [50]	+	-	-	-	-	+	-	-	25.0%
Reinking et al. [51]	+	-	-	-	+	+	+	-	50.0%
Finnoff et al. [52]	-	+	-	+	-	+	+	+	62.5%
Bennett et al. [53]	-	-	-	-	-	-	+	-	12.5%
**Road: long-distance runners**									

Koplan et al. [23]	+	-	-	-	+	-	+	-	37.5%
Nicholl and Williams [34]	-	-	-	-	+	-	-	+	25.0%
Nicholl and Williams [35]	+	-	+	-	+	+	-	+	62.5%
Hughes et al. [54]	+	-	-	-	+	+	+	+	62.5%
Jacobs and Berson [55]	-	-	-	-	+	+	+	-	37.5%
Lloyd et al. [56]	-	+	-	-	-	-	+	-	25.0%
Marti [58]	-	-	+	+	+	+	+	-	62.5%
Marti et al. [57]	+	-	+	+	+	+	+	-	75.0%
Koplan et al. [3]	+	+	-	-	+	-	+	-	50.0%
Yeung et al. [36]	+	-	+	-	+	+	-	+	62.5%
Woolf et al. [59]	+	+	-	-	-	-	-	-	25.0%
Micklesfield et al. [37]	-	+	-	-	-	-	-	-	12.5%
Chang et al. [38]	-	+	-	-	-	-	-	-	12.5%
Pasquina et al. [60]	+	-	+	-	+	+	-	+	62.5%
**Marathon runners**									
Caldwell [61]	-	-	-	-	-	-	+	+	25.0%
Maughan and Miller [62]	-	-	-	-	-	-	-	-	0.0%
Kretsch et al. [63]	-	-	-	-	+	-	-	+	25.0%
McKelvie et al. [64]	-	+	-	-	+	-	-	-	25.0%
Hölmich et al. [65]	+	-	+	+	+	-	-	+	62.5%
Hölmich et al. [66]	-	+	-	-	-	-	-	-	12.5%
Macera et al. [67]	-	-	-	-	+	+	+	+	50.0%
Jakobsen et al. [68]	+	-	-	+	+	-	+	+	62.5%
Satterthwaite et al. [69]	+	-	+	+	+	+	-	+	75.0%
Satterthwaite et al. [96]	-	-	+	+	+	+	-	+	62.5%
Roberts et al. [70]	+	-	+	-	+	+	+	+	75.0%
van Middelkoop et al. [71]	+	+	-	+	+	-	+	+	75.0%
van Middelkoop et al. [72]	-	+	-	+	+	-	+	+	62.5%
Parker et al. [73]	-	+	-	-	+	-	-	+	37.5%
Rasmussen et al. [74]	-	+	-	-	+	+	+	+	62.5%
Ogwumike and Adeniyi [97]	+	-	+	-	+	+	-	+	62.5%
**Ultra-marathon runners**									
Hutson [75]	-	-	+	+	+	+	-	+	62.5%
Fallon [76]	-	-	+	+	-	+	-	+	50.0%
Bishop and Fallon [77]	-	-	+	+	+	+	+	+	75.0%
Krabak et al. [78]	+	+	+	+	+	+	+	+	100.0%
Scheer and Murray [79]	-	-	+	+	+	+	-	+	62.5%
Hoffman and Krishnan [80]	-	-	-	-	+	-	+	-	25.0%
