# Supplementary material for: What are the Differences in Injury Proportions Between Different Populations of Runners? A Systematic Review and Meta-Analysis
Source: Sports Med. 2015 Apr 8;45(8):1143–61. doi: 10.1007/s40279-015-0331-x (PMC4513221; doi:10.1007/s40279-015-0331-x)

Electronic Supplementary Material Appendix S5. Sensitivity analysis on the low risk of bias studies (ROB score ≥ 50)

Medical attention injuries during an event

Study	Design	Injuries	Participants	Injury proportion (%)	95% CI
**Population = Track: sprinters**					
Alonso et al. (2009) [29]	PC	17	327	5.2	[3.1, 8.2]
Alonso et al. (2012) [31]	PC	31	324	9.6	[6.6, 13.3]
**Random effects model**		**48**	**651**	**7.2**	**[3.9, 12.9]**
Heterogeneity: I^2=77.4%, τ^2=0.1671, p=0.0355					
**Population = Track: middle distance runners**					
Alonso et al. (2009) [29]	PC	16	172	9.3	[5.4, 14.7]
Alonso et al. (2012) [31]	PC	26	154	16.9	[11.3, 23.8]
**Random effects model**		**42**	**326**	**12.8**	**[7.0, 22.3]**
Heterogeneity: I^2=75.3%, τ^2=0.1759, p=0.0441					
**Population = Track: long distance runners**					
Alonso et al. (2009) [29]	PC	17	101	16.8	[10.1, 25.6]
Alonso et al. (2012) [31]	PC	15	105	14.3	[8.2, 22.5]
**Random effects model**		**32**	**206**	**15.6**	**[11.2, 21.2]**
Heterogeneity: I^2=0%, τ^2=0, p=0.6144					
**Population = Road: long distance runners**					
Nicholl and Williams (1983) [35]	PC	41	1,140	3.6	[2.6, 4.8]
Yeung et al. (1998) [36]	PC	25	4,600	0.5	[0.4, 0.8]
Pasquina et al. (2013) [60]	PC	346	91,750	0.4	[0.3, 0.4]
**Random effects model**		**412**	**97,490**	**0.9**	**[0.2, 3.8]**
Heterogeneity: I^2=98.9%, τ^2=1.624, p<0.0001					
**Population = Marathon runners**					
Nicholl and Williams (1983) [35]	PC	409	2,289	17.9	[16.3, 19.5]
Satterthwaite et al. (1996) [69], (1999) [96]	PC	75	1,219	6.2	[4.9, 7.7]
Yeung et al. (1998) [36]	PC	46	900	5.1	[3.8, 6.8]
Roberts (2000) [70]	PC	1,459	76,714	1.9	[1.8, 2.0]
Alonso et al. (2009) [29]	PC	20	151	13.2	[8.3, 19.7]
Alonso et al. (2012) [31]	PC	28	124	22.6	[15.6, 31.0]
Ogwumike and Adeniyi (2013) [97]	PC	153	920	16.6	[14.3, 19.2]
**Random effects model**		**2,190**	**82,317**	**9.4**	**[3.3, 23.9]**
Heterogeneity: I^2=99.7%, τ^2=2.207, p<0.0001					
**Population = Ultra marathon runners**					
Hutson (1984) [75]	PC	15	25	60.0	[38.7, 78.9]
Fallon (1996) [76]	PC	29	32	90.6	[75.0, 98.0]
Bishop and Fallon (1999) [77]	PC	11	17	64.7	[38.3, 85.8]
Krabak et al. (2011) [78]	PC	257	396	64.9	[60.0, 69.6]
Scheer and Murray (2011) [79]	PC	39	69	56.5	[44.0, 68.4]
**Random effects model**		**351**	**539**	**65.6**	**[55.6, 74.4]**
Heterogeneity: I^2=58.9%, τ^2=0.1173, p=0.045					

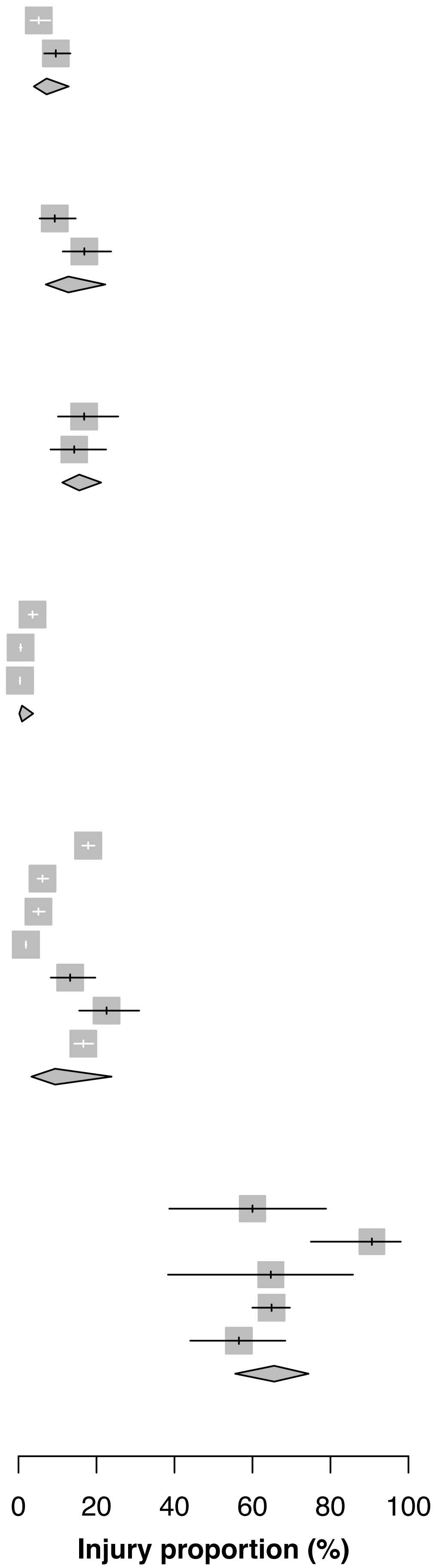

Time-loss injuries during a short follow-up/recall period

Study	Design	Injuries	Participants	Injury proportion (%)	95% CI
**Population = Novice runners**					
Buist et al. (2008) [82], (2010) [83]	PC	100	486	20.6	[17.1, 24.4]
van Ginckel et al. (2009) [86]	PC	69	129	53.5	[44.5, 62.3]
Bredeweg et al. (2012) [88], (2013) [89], (2013) [90]	PC	58	362	16.0	[12.4, 20.2]
Nielsen et al. (2013) [92]	PC	13	58	22.4	[12.5, 35.3]
**Random effects model**		**240**	**1,035**	**26.4**	**[14.2, 43.7]**
Heterogeneity: I^2=95.7%, τ^2=0.5871, $p<0.0001$					
**Population = Recreational runners**					
Buist et al. (2010) [7]	PC	163	629	25.9	[22.5, 29.5]
Hespanhol Junior et al. (2013) [95]	PC	60	191	31.4	[24.9, 38.5]
**Random effects model**		**223**	**820**	**28.0**	**[23.1, 33.5]**
Heterogeneity: I^2=55.2%, τ^2=0.02, $p=0.1353$					
**Population = Cross–country runners**					
Garrick and Requa (1978) [20], (1978) [21]	PC	50	167	29.9	[23.1, 37.5]
Chandy and Grana (1985) [5]	PC	31	2,278	1.4	[0.9, 1.9]
Beachy et al. (1997) [16]	PC	197	1,288	15.3	[13.4, 17.4]
Rauh et al. (2000) [42]	PC	927	3,233	28.7	[27.1, 30.3]
Rauh et al. (2006) [44], (2007) [54]	PC	162	421	38.5	[33.8, 43.3]
Reinking (2006) [6]	PC	3	18	16.7	[3.6, 41.4]
Reinking et al. (2010) [51]	PC	45	93	48.4	[37.9, 59.0]
**Random effects model**		**1,415**	**7,498**	**19.7**	**[10.9, 33.1]**
Heterogeneity: I^2=98.7%, τ^2=0.8301, $p<0.0001$					

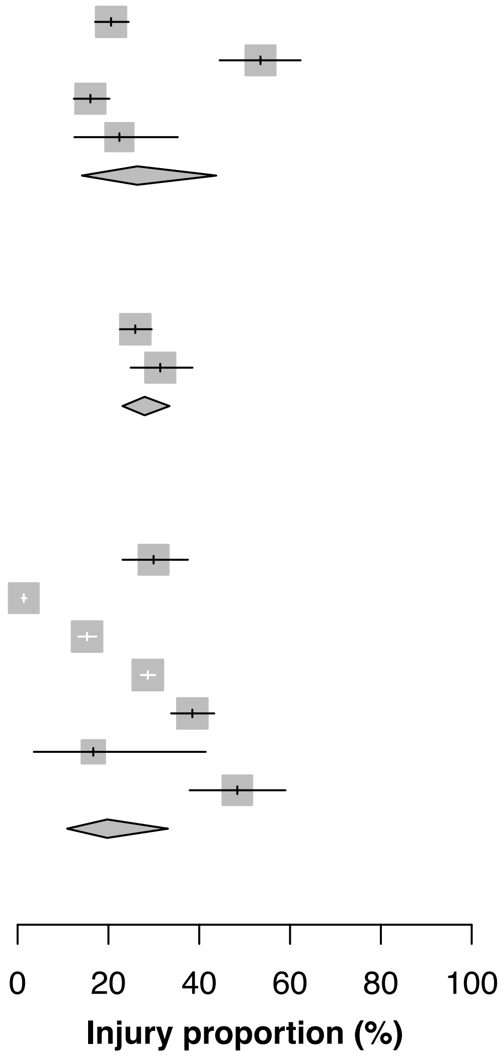

Pain-related injuries during a short follow-up/recall period

Study	Design	Injuries	Participants	Injury proportion (%)	95% CI
**Population = Recreational runners**					
Buist et al. (2010) [7]	PC	217	629	34.5	[30.8, 38.4]
**Random effects model**		**217**	**629**	**34.5**	**[30.9, 38.3]**
Heterogeneity: not applicable for a single study					
**Population = Cross–country runners**					
Beachy et al. (1997) [16]	PC	610	1,288	47.4	[44.6, 50.1]
Reinking (2006) [6]	PC	9	18	50.0	[26.0, 74.0]
**Random effects model**		**619**	**1,306**	**47.4**	**[44.7, 50.1]**
Heterogeneity: I^2=0%, τ^2=0, p=0.8238					

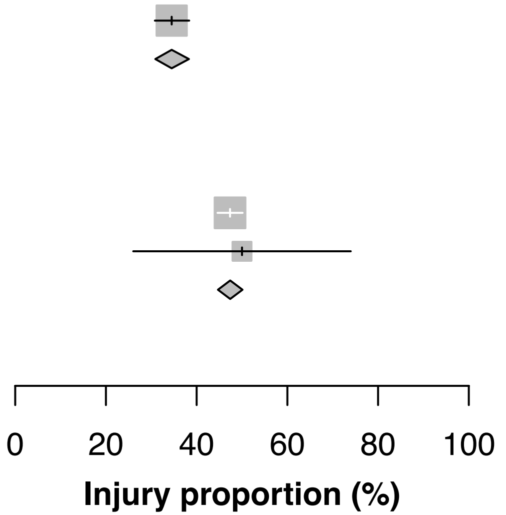

Time-loss injuries during a 1-year follow-up/recall period

Study	Design	Injuries	Participants	Injury proportion (%)	95% CI
**Population = Track: sprinters**					
Lysholm and Wiklander (1987) [27]	PC	13	19	68.4	[43.4, 87.4]
Yeung et al. (2009) [40]	PC	25	44	56.8	[41.0, 71.7]
Jacobsson et al. (2013) [41]	PC	50	77	64.9	[53.2, 75.5]
**Random effects model**		**88**	**140**	**62.8**	**[54.4, 70.4]**
Heterogeneity: I^2=0%, τ^2=0, p=0.5839					
**Population = Track: middle distance runners**					
Lysholm and Wiklander (1987) [27]	PC	10	13	76.9	[46.2, 95.0]
**Random effects model**		**10**	**13**	**76.9**	**[47.8, 92.4]**
Heterogeneity: not applicable for a single study					
**Population = Novice runners**					
Nielsen et al. (2013) [25], (2013) [91]	PC	254	930	27.3	[24.5, 30.3]
**Random effects model**		**254**	**930**	**27.3**	**[24.5, 30.3]**
Heterogeneity: not applicable for a single study					
**Population = Recreational runners**					
Hespanhol Junior et al. (2012) [94]	RC	110	200	55.0	[47.8, 62.0]
**Random effects model**		**110**	**200**	**55.0**	**[48.1, 61.8]**
Heterogeneity: not applicable for a single study					
**Population = Cross–country runners**					
Shively et al. (1981) [26]	PC	9	576	1.6	[0.7, 2.9]
McLain and Reynolds (1989) [24]	PC	10	94	10.6	[5.2, 18.7]
**Random effects model**		**19**	**670**	**4.2**	**[0.6, 23.9]**
Heterogeneity: I^2=94.5%, τ^2=1.917, p<0.0001					
**Population = Road: long distance runners**					
Marti et al. (1988) [57], (1988) [58]	RC	1,372	4,786	28.7	[27.4, 30.0]
**Random effects model**		**1,372**	**4,786**	**28.7**	**[27.4, 30.0]**
Heterogeneity: not applicable for a single study					
**Population = Marathon runners**					
Hölmich et al. (1988) [65]	RC	26	60	43.3	[30.6, 56.8]
Macera et al. (1991) [67]	RC	85	162	52.5	[44.5, 60.4]
Jakobsen et al. (1994) [68]	PC	31	41	75.6	[59.7, 87.6]
van Middelkoop et al. (2008) [71], (2008) [72]	PC	397	725	54.8	[51.1, 58.4]
Rasmussen et al. (2013) [74]	RC	273	662	41.2	[37.5, 45.1]
**Random effects model**		**812**	**1,650**	**52.0**	**[43.1, 60.8]**
Heterogeneity: I^2=89.3%, τ^2=0.1312, p<0.0001					

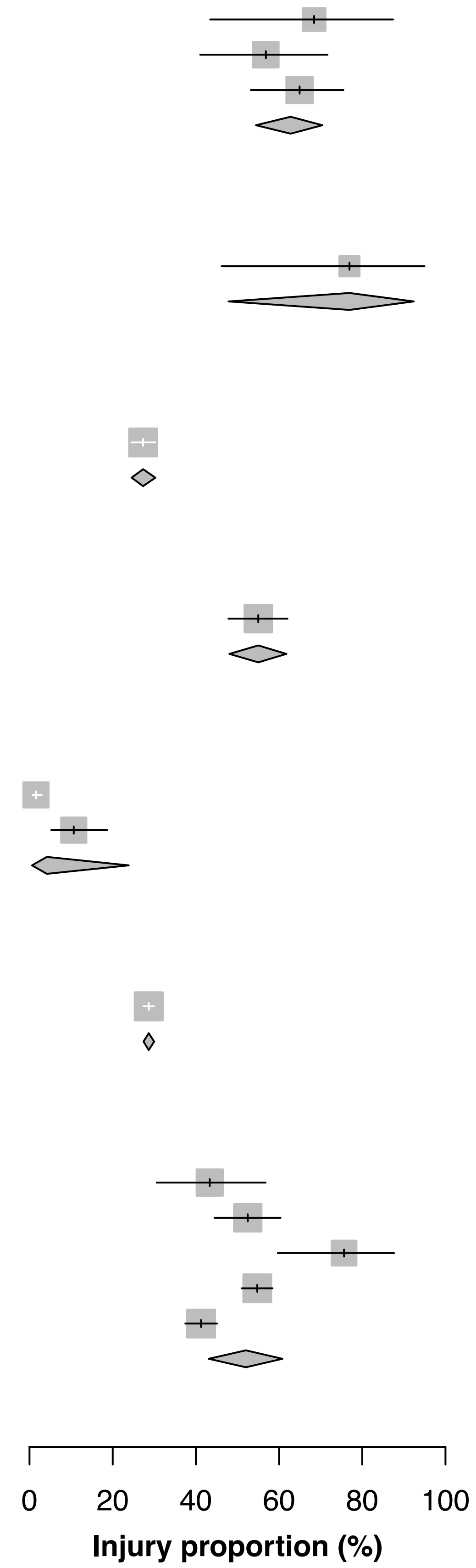

Time-loss injuries during a long follow-up/recall period

Study	Design	Injuries	Participants	Injury proportion (%)	95% CI
**Population = Novice runners**					
Bovens et al. (1989) [81]	PC	62	73	84.9	[74.6, 92.2]
**Random effects model**		**62**	**73**	**84.9**	**[74.8, 91.5]**
Heterogeneity: not applicable for a single study					
**Population = Cross–country runners**					
Reinking (2006) [6]	RC	12	18	66.7	[41.0, 86.7]
Reinking et al. (2010) [51]	RC	103	125	82.4	[74.6, 88.6]
**Random effects model**		**115**	**143**	**77.4**	**[60.6, 88.4]**
Heterogeneity: I^2=57.8%, τ^2=0.2091, p=0.1236					
**Population = Road: long distance runners**					
Koplan et al. (1995) [3]	RC	281	535	52.5	[48.2, 56.8]
**Random effects model**		**281**	**535**	**52.5**	**[48.3, 56.7]**
Heterogeneity: not applicable for a single study					

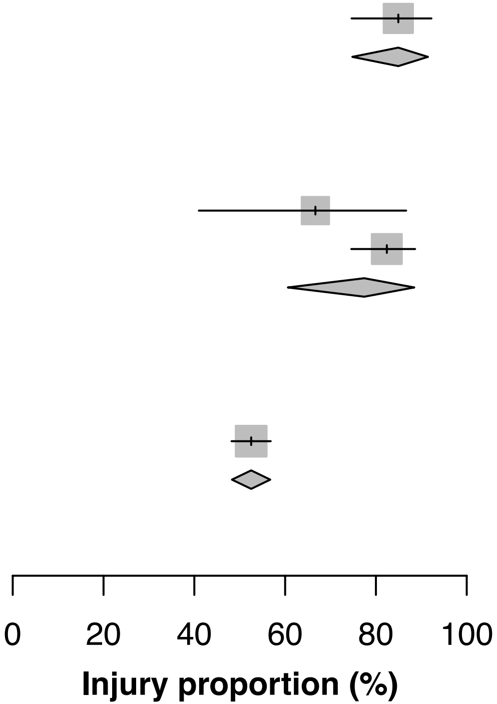

Supplement: Supplementary file 5 — Supplementary material 5 (PDF 618 kb) [file 40279_2015_331_MOESM5_ESM.pdf]
