# Supplementary material for: What are the Differences in Injury Proportions Between Different Populations of Runners? A Systematic Review and Meta-Analysis
Source: Sports Med. 2015 Apr 8;45(8):1143–61. doi: 10.1007/s40279-015-0331-x (PMC4513221; doi:10.1007/s40279-015-0331-x)
Supplement: Supplementary file 6 — Supplementary material 6 (PDF 80 kb) [file 40279_2015_331_MOESM6_ESM.pdf]

Electronic Supplementary Material Appendix S6. Site-specific injury proportions (%) for each population of runners

Study	Population	Time period	Injury definition	Foot	Ankle	Lower leg	Knee	Upper leg	Hip/pelvis	LEX not possible to categorize	Not LEX	Other sites, not stated	Number of injuries
D'Souza [18]	Track sprint	None	TL	22.2	0.0	18.5	7.4	37.0	11.1		29.6		27
Jacobsson et al. [41]	Track sprint	Year	TL					32.0	10.7	53.3	4.1		122
D'Souza [18]	Track middle	Year	TL	6.7	20.0	46.7	20.0	13.3	6.7		13.4		15
Bovens et al. [81]	Novice	Long	TL	5.7	12.1	32.2	24.7	6.3	11.5		7.5		174
Buist et al. [82, 83]	Novice	Short	TL			40.0	37.0	4.0	9.0	10.0			100
van Ginckel et al. [86]	Novice	Short	TL	2.9	10.1	33.3	36.2	5.8	8.7		2.9		69
Buist et al. [7]	Recreational	Short	TL	3.7	5.5	33.7	29.4	3.1	8.0		4.9	11.7	163
Lopes et al. [93]	Recreational	None	PRI				27.8	8.8	11.0	30.8	12.8	8.8	227
Hespanhol Junior et al. [94]	Recreational	Year	TL	14.5	11.8	20.0	27.3	8.2	9.1		9.1		110
Hespanhol Junior et al. [95]	Recreational	Short	TL	16.7	7.1	3.6	19.0	14.3	8.4	14.3	16.7		84
Rauh et al. [42]	Cross-country	Short	TL	9.6	19.2	29.2	22.0	7.4	6.8		3.4	2.4	795
Rauh et al. [44, 45]	Cross-country	Short	TL			36.0	25.3	17.3		18.0	3.3		148
Hughes et al. [54]	Road long	Event	TL	14.7	8.6	16.9	35.2	9.2	10.0		5.5		361
Jacobs and Berson [55]	Road long	Long	TL	7.7	18.3	32.5	31.7	9.9					142
Yeung et al. [36]	Road long	Event	MA	10.0	3.3		23.3	30.0		26.7	6.7		30
Caldwell [61]	Marathon	Event	TL	30.9	1.8	21.8	27.3	7.3	9.1		1.8		55
Maughan and Miller [62]	Marathon	Short	TL	13.4	12.0	23.5	31.5	8.6	5.3		4.9	0.8	358
Satterthwaite et al. [69, 96]	Marathon	Event	PRI	22.1		23.4	12.7	41.8					1,812
Satterthwaite et al. [69, 96]	Marathon	Short	PRI	11.9		25.1	15.4	47.7					1,425
Yeung et al. [36]	Marathon	Event	MA	2.8	7.0		9.9	33.8		43.7	2.8		71
van Middelkoop et al. [71]	Marathon	Event	TL	21.4	3.3	34.0	21.3	14.0	6.0				150
van Middelkoop et al. [71]	Marathon	Year	TL	14.3	6.0	35.6	22.2	8.9	12.9				550
Ogwumike and Adeniyi [97]	Marathon	Event	MA	14.2	17.7	17.7	13.8	31.9	4.3		0.4		254
Rasmussen et al. [74]	Marathon	Year	TL			19.1	32.4	5.9	2.9	32.4	4.4	2.9	68
Fallon [76]	Ultra marathon	Event	MA	6.3	28.1	14.1	31.3	10.9			9.5		64
Bishop and Fallon [77]	Ultra marathon	Event	MA	5.6	36.1	11.1	19.4	11.1	11.1		5.6		36
Scheer and Murray [79]	Ultra marathon	Event	MA		27.3	13.6	40.9	4.5	13.6				22

TL: time-loss injury, MA: medical attention injury, PRI: pain-related injury, LEX: lower extremity
